# Bispecific antibodies targeting EGFR/Notch enhance the response to talazoparib by decreasing tumour-initiating cell frequency

**DOI:** 10.7150/thno.82144

**Published:** 2023-06-26

**Authors:** Wenyan Fu, Guangyao Li, Changhai Lei, Kewen Qian, Shuyi Zhang, Jian Zhao, Shi Hu

**Affiliations:** 1 Department of Assisted Reproduction, Shanghai Ninth People's Hospital, Shanghai Jiao Tong University School of Medicine, Shanghai 200000, China.; 2 Department of Biomedical Engineering, College of Basic Medical Sciences, Naval Medical University (Second Military Medical University), Shanghai, China; 3 Department of Biophysics, College of Basic Medical Sciences, Naval Medical University (Second Military Medical University), Shanghai, China; 4 KOCHKOR Biotech, Inc., Shanghai, Shanghai 201406, China

**Keywords:** PARP, EGFR, Cancer stem cell, Notch, BsAb

## Abstract

Poly ADP ribose polymerase (PARP) inhibitors are mainly used in treating BRCA-mutant cancers, and their application in novel therapies to expand their benefit is of interest in personalized medicine. A recent report showed that pharmacological targeting of PARP increases the sensitivity of cancer cells to EGFR inhibition, but the therapeutic value of this combination has not been fully determined. We propose a strategy of combining PARP inhibitors with bispecific antibodies that target both EGFR and Notch signalling, highlighting the difficulties posed by deregulation of Notch signalling and the enrichment of cancer stem cells (CSCs) during therapy. In the present study, we showed that although PARP plus EGFR targeting led to more penetrant and durable responses in the non-small cell lung cancer (NSCLC) PDX model, it influenced the enrichment of stem-like cells and their relative proportion. Stem-like cells were significantly inhibited in vitro and in vivo by EGFR/Notch-targeting bispecific antibodies. These bispecific antibodies were effective in PDX models and showed promise in cell line models of NSCLC, where they delayed the development of acquired resistance to cetuximab and talazoparib. Moreover, combining EGFR/Notch-targeting bispecific antibodies and talazoparib had a more substantial antitumour effect than the combination of talazoparib and cetuximab in a broad spectrum of epithelial tumours. EGFR/Notch bispecific antibodies decrease the subpopulation of stem-like cells, reduce the frequency of tumour-initiating cells, and downregulate mesenchymal gene expression. These findings suggest that combining EGFR and Notch signalling blockade can potentially increase the response to PARP blockade.

## Introduction

Overexpression or abnormal activation of the epidermal growth factor receptor (EGFR) is observed in many human cancers 1-3. Numerous human EGFR-targeting agents, such as monoclonal antibodies (such as cetuximab and panitumumab) and third-generation EGFR tyrosine kinase inhibitors (TKIs), have been approved by the Food and Drug Administration (FDA) due to their excellent clinical performance. In various cancer models, targeted therapy using monoclonal antibodies efficiently prevents EGFR ligand binding, and receptor dimerization promotes EGFR phosphorylation and internalization, which ultimately reduces cell proliferation 4. The P27KIP1-CDK2 complex is ultimately prevented from exiting G1 phase by cetuximab, which also causes the arrest of the G1 phase of the cell cycle and increases P27KIP1 induction levels. Numerous studies have demonstrated that cetuximab treatment can reduce tumour survival through a number of mechanisms, including downregulating angiogenic factors and matrix metalloproteinases that are involved in cell adhesion to lessen cancer cell metastasis, upregulating Bax and other proapoptotic factors, downregulating Bcl2 and activating caspases, and inducing ADCC in vivo by attracting immune cells to tumour cells 5. However, although TKIs and EGFR-targeting antibodies are frequently used in the clinical treatment of non-small cell lung cancer (NSCLC), EGFR-targeting therapy faces a major challenge because patients often develop innate or acquired drug resistance within a year of starting treatment 6, 7.

The tumour-initiating cell (TIC) or cancer stem cell (CSC) theory has drawn much interest. According to this theory, a small subpopulation of cancer cells that resemble stem cells can self-renew and differentiate at the top of the tumour cell hierarchy 8. Stem cell-like cancer cells are thought to play a role in tumour progression, recurrence, and resistance to chemotherapy and radiation therapy 9, indicating their status as an important target in cancer therapy. In addition to having greater resistance to paclitaxel and docetaxel than their parental cells, cell lines that are resistant to EGFR inhibitors also have a higher capacity to initiate tumours. These characteristics might be connected to the growth of CSC subsets 10. In addition, one study showed that CSCs have an inherent resistance to EGFR blockade 11. Another study reported the activation of EGFR-dependent Notch3 signalling after erlotinib treatment of lung cancer cell lines with EGFR mutations, which is responsible for the enrichment of stem cell-like cancer cells 12. These results support the data from recent clinical trials of EGFR blockers, in which EGFR blockade failed to improve survival after the patients had received curative-intent therapy in the early stage of the disease 13, 14. Moreover, although controversial, increasing evidence suggests that CSCs have a higher baseline DNA damage response (DDR) and single-strand break response (SSBR), including after ionizing radiation, which contributes to the enhanced tolerance of CSCs to DNA damage stress and oxidative stress 15, 16.

The enzyme poly-ADP-ribose polymerase 1 (PARP1) catalyses the transfer of ADP-ribose polymers to substrates. PARP1 can sense DNA lesions, activate DDRs and act as a DNA damage repair enzyme. As a key player in single-strand break repair and the DNA damage response, PARP has been identified as a potential therapeutic target. In preclinical research and clinical trials, numerous PARP inhibitors have been used to treat various cancer types, including NSCLC 17. The efficacy of PARP inhibitors combined with TMZ has been validated at the clinical level. In a phase I trial and a phase II/III trial, olaparib and veliparib, two PARP inhibitors, were used, respectively. However, excessive toxicity reduced their effectiveness and resulted in the failure of clinical trials 18, 19. A novel oral PARP inhibitor called talazoparib is currently being developed and has demonstrated greater in vitro activity than any other PARP inhibitor 20, 21. Treatment with talazoparib demonstrated promising single-agent lethality in advanced ovarian and breast cancer patients carrying harmful BRCA1/2 mutations. However, the rarity of BRCA mutations in other cancer types, including NSCLC 22, has limited the wide application of talazoparib. Talazoparib is currently being tested in a clinical study for NSCLC (NCT04173507). Investigation of new methods to increase the proportion of NSCLC patients who are anticipated to benefit from talazoparib treatment is urgently needed.

EGFR blockade downregulates key players in base excision repair (BER) and increases the sensitization of cancer cells to alkylating agents and ionizing radiation 23, 24. Previous studies have shown that EGFR-mutant cancer cells are sensitive to olaparib both in vitro and in vivo 25. Recent studies have demonstrated that overexpressing EGFR is linked to an increase in reactive oxygen species (ROS), which is followed by an upregulation of DNA damage repair pathway basal expression to combat rising oxidative stress, decreasing cell sensitivity to PARP inhibitors 26. Moreover, a recent study showed that pharmacological targeting of PARP led to enhanced sensitivity of cancer cells to EGFR inhibition.

The cross-regulation of EGFR and Notch signalling in embryonic development has long been shown by genetic studies. These interactions can appear antagonistic or constructive depending on the animal model or organ under investigation 27, 28. Notch receptors are primordial, evolutionarily conserved, and highly associated with CSCs 29. The diversity of Notch receptors and ligands, with four paralogous Notch receptors (Notch1 to Notch4) and five canonical Notch ligands [Delta-like ligand 1 (DLL1), DLL3, DLL4, Jagged1, and Jagged2] identified thus far, is the basis for the complex and versatile functional activities of Notch signalling. The resistance of cancer cells to molecularly targeted therapies, promotion of the epithelial-mesenchymal transition (EMT), and increased invasion of cancer cells are all caused by the crosstalk between Notch and EGFR signalling 30. In addition, EGFR was reported to have a kinase-dependent physical association with the Notch receptor, with the ability to modulate the functional activity of Notch signalling in human lung cancer cell lines 12. Additionally, EGFR blockade was demonstrated to enhance Notch expression and the EMT process, increasing the frequency of cancer-associated fibroblasts (CAFs)^31^. Additionally, we recently reported that bispecific antibodies reduced the frequency of CSCs in preclinical models by simultaneously inhibiting EGFR and Notch signalling 32, 33. Recently, NOTCH3 was shown to send a signal via β-catenin instead of the normal notch pathway. By binding to β-catenin, NOTCH3 stabilizes the transcription factor and induces β-catenin-dependent gene expression 34.

These results provide a rationale for cotargeted therapies based on EGFR, PARP, and Notch signalling in cancer and suggest a direct interaction among these signalling pathways. Therefore, we argue that a PARP inhibitor and a combination of EGFR and Notch signalling blockade would be most effective against NSCLC.

## Materials and Methods

### Cell lines and reagents

HCC2279 was obtained from the Korean Cell Line Bank, and H322 was obtained from the European Collection of Cell Cultures; all other cell lines were purchased from the American Type Culture Collection (ATCC). Using STR analysis, we were able to confirm the purity of all of the cell lines and ensure that they were true to type. The cells were cultured in accordance with protocols developed by the ATCC. We ordered talazoparib from Selleck and cetuximab from Merck. As we previously reported, we produced BsAb CT16 and PTJ12 in-house.

### Aldefluor assay and flow cytometry

The validation of ALDH+ cells was performed using an Aldefluor Assay Kit from Stemcell Technologies. The specific assay procedure was carried out in accordance with the manufacturer's operating guidelines, with the necessary modifications. Phycoerythrin-conjugated anti-human CD133/1 antibody (Miltenyi Biotec) was used to stain the assayed cells. The phycoerythrin-conjugated anti-human CD133/1 antibody was tested against an isotype control antibody. The ALDEFLUOR assay's negative control was the specific ALDH inhibitor diethylaminobenzaldehyde, which can establish the baseline fluorescence of these cells. With a BD FACSVantage SE cell sorter and CellQuest software (BD Biosciences), all cell samples were examined and sorted. For analysis of the purity of the cells, aliquots of CD133+ALDH-, CD133+ALDH+, CD133-ALDH+, and CD133-ALDH- sorted cells were costained with phycoerythrin-conjugated anti-human CD133/2 antibody (Miltenyi Biotec) and examined using a FACSCalibur flow cytometer (BD Biosciences).

### Protein array

According to the manufacturer's instructions, an antibody-based custom protein Array (Sinobiological, Beijing, China) was used to analyze the expression of EMT markers and signalling. Overnight at 4°C, cell lysates were centrifuged and hybridized to the array membrane. After washing the membrane, a second antibody against the protein (biotin-conjugated) was applied, and HRP-conjugated streptavidin was pipetted onto the membrane to detect the proteins.

### Cell proliferation assay

For calculation of the IC50, cells were plated in triplicate in 96-well plates at 5 x 10^3^ cells per well and cultured for an overnight period. The cell culture medium of plated cells was replaced with RPMI medium containing 0.2% foetal bovine serum (assay medium) and DMEM with or without treatment agents. Multiple concentrations of talazoparib and cetuximab (maximal doses of 50 mM and 100 mg/ml, respectively) were added to the cell culture medium. With a CellTiter-Glo Luminescent Cell Viability Assay from Promega, cell viability was evaluated 72 hours after treatment. The results were obtained from a minimum of three repeated experiments, and the drug concentrations that resulted in a 50% reduction in cell viability (IC50) were calculated based on a four-variable curve analysis.

Cancer cells (2000-5000 cells per well) were plated in 96-well plates for the cell proliferation assay. The following day, the cells were treated with the indicated antibody concentrations in medium containing 5% serum. After 4-6 days, alamarBlue (Invitrogen) was added to the wells. The fluorescence in the 96-well plate was detected with excitation at 530 nm and emission at 590 nm. Relative fluorescence units (RFU) are used to express the results.

### Real-time polymerase chain reaction (PCR)

Total RNA was isolated using a Qiagen RNeasy Mini Kit. The data were then analysed with SDS v2.3 software (Applied Biosystems) after real-time quantitative PCR was carried out with an ABI Prism 7900HT system. Actin expression was used as an endogenous control for typical expression. Commercial TaqMan probes were used for real-time quantitative PCR with the following assay IDs: EGFR (Hs01076078), HER3 (Hs00176538), CDH1 (Hs01023895), CDH2 (Hs00983062), CLDN3 (Hs00265816), CLDN7 (Hs00600772), CLDN12 (Hs00273258), CRB3 (Hs01548179), DIGH1 (Hs00938192), FN1 (Hs01549976), HES1 (Hs00172878), HEY1 (Hs01114113), INADL (Hs00195106), JAG1 (Hs01070032), NOTCH1 (Hs01062014), NOTCH2 (Hs01050702), NOTCH3 (Hs01128537), NOTCH4 (Hs00965889), SCRIB (Hs00363005), SNAI1 (Hs00195591), SNAI2 (Hs00161904), TWIST1 (Hs00361186), TWIST2 (Hs002379), VIM (Hs00958111), ZEB1 (Hs01566408), and ZEB2 (Hs00207691). The real-time quantitative PCR data were analysed and log transformed to generate a heatmap 35.

### Immunofluorescence staining

Cells were plated on cover slides, fixed with 4% paraformaldehyde, permeabilized with 0.3% Triton X-100, and blocked with 1% bovine serum albumin prior to immunofluorescence staining. After an overnight incubation, the primary antibodies were labeled with the corresponding fluorescent dye-conjugated secondary antibody. The primary antibodies were incubated. DAPI was used to stain the cell nuclei. A Leica TCS SP2 confocal system from Germany's Leica was utilized in order to perform observations and capture images of the samples.

### siRNA transfection

Pools of siRNAs directly targeting Notch receptors and nontargeting siRNAs were purchased from Dharmacon. Dharma-FECT 4 transfection reagent (Dharmacon) was used for transfection of cells with individual siRNAs at a dose of 2 nmol.

### In vivo study of cancer cell line therapy

All in vivo experiments were approved by the Institutional Animal Care and Use Committee (IACUC) of Second Military Medical University, and the mice were housed in a specific pathogen-free barrier facility. NSCLC cells were injected into BALB/c nude mice to create the NSCLC tumour model (Shanghai Experimental Animal Center of Chinese Academy of Sciences). The mice were randomly divided into corresponding groups with 8-12 mice in each group when the tumour volumes averaged approximately 150 mm^3^. A 2x loading dose served as the first dose (the dose given on the day of randomization), and multiple dose studies continued throughout the course of the 4 weeks of treatment. Throughout the course of the study, tumours were measured with digital callipers at least once per week, and the volume of each tumour was calculated using the formula volume = length × (width) ^2^/2.

### Patient-derived xenograft models

Tumour specimens were obtained during the initial surgery on patients with newly diagnosed NSCLC in Changzheng Hospital of Naval Medical University. The acquisition of the samples was based on written informed consent by each patient and was ethically certified by hospital pathologists. As previously described, a minimal initiating tumour cell frequency and human tumour xenograft models minimally passaged were established (*34, 53*). Briefly, human tumour xenograft models were established by subcutaneous implantation of patient-derived solid tumour fragments in NOD/SCID mice (Animal Center of Chinese Academy of Sciences). All animal manipulations were carried out in accordance with the requirements established by the Committee on Animals of the Naval Medical University. Specific doses of drugs were administered to mice via intraperitoneal injection at certain dosing intervals. Radiation treatment was performed using a cabinet X-ray biological irradiator X-RAD 320 from the Radiology Department of the PLA General Hospital. The mice were immobilized with custom-designed lead jigs, and their tumour-bearing back was then exposed to radiation, avoiding irradiation of nontumour-bearing normal tissue as much as possible. The body condition index 36, animal weight, skin appearance, and posture were all monitored to evaluate toxicity. The established tumours were isolated and prepared into single-cell suspensions, which were frozen at -80 °C for the construction of tumour models required for subsequent experiments. Single-cell suspensions from untreated or treated tumours were prepared for the tumorigenicity assay and cell-sorting experiments. These suspensions were then incubated on ice for 30 min with biotinylated mouse CD45 and mouse H2Kd antibodies. Magnetic beads that were dyed with streptavidin were then added to remove the murine stromal cells. After being collected, human tumour cells were counted and diluted for FACS analysis or subcutaneous injection into NOD/SCID mice. Up to three months of tumour growth was observed. The L-Calc Version 1.1 software program (StemCell Technologies) was used to calculate the frequency of cancer stem cells. All therapeutic agents were intraperitoneally administered.

### Tissue analysis

Whole excised tumour tissue was lysed using the Sigma CelLytic MT Lysis Reagent, and total RNA was isolated using the Qiagen RNeasy Mini Kit in accordance with the manufacturer's instructions. On an ABI Prism 7900HT system from Applied Biosystems, real-time quantitative PCR was carried out using Solaris qPCR GENE Expression Assays SYBR from Thermo Fischer Scientific.

### In vivo studies of tumorigenicity

SCID mice were subcutaneously injected with various numbers of tumour cells suspended in Matrigel (BD Biosciences) at a ratio of 1:1. The mice were killed between three and six months after the initial injection, when the tumours had developed. Five months after the inoculation of tumour cells, mice with injected tumours but without s significant tumour burden were typically put to sleep. During this time, the injection sites were surgically examined to ensure that no tumour had formed.

### Statistical analysis

Unless otherwise specified, Student's t test was used to evaluate significant differences between 2 groups, and ANOVA was used to evaluate differences among 3 or more quantitative groups. Differences between groups were considered statistically significant when *P* < 0.05.

## Results

### EGFR-targeting antibody enhances the response to talazoparib

**Figure 1:**
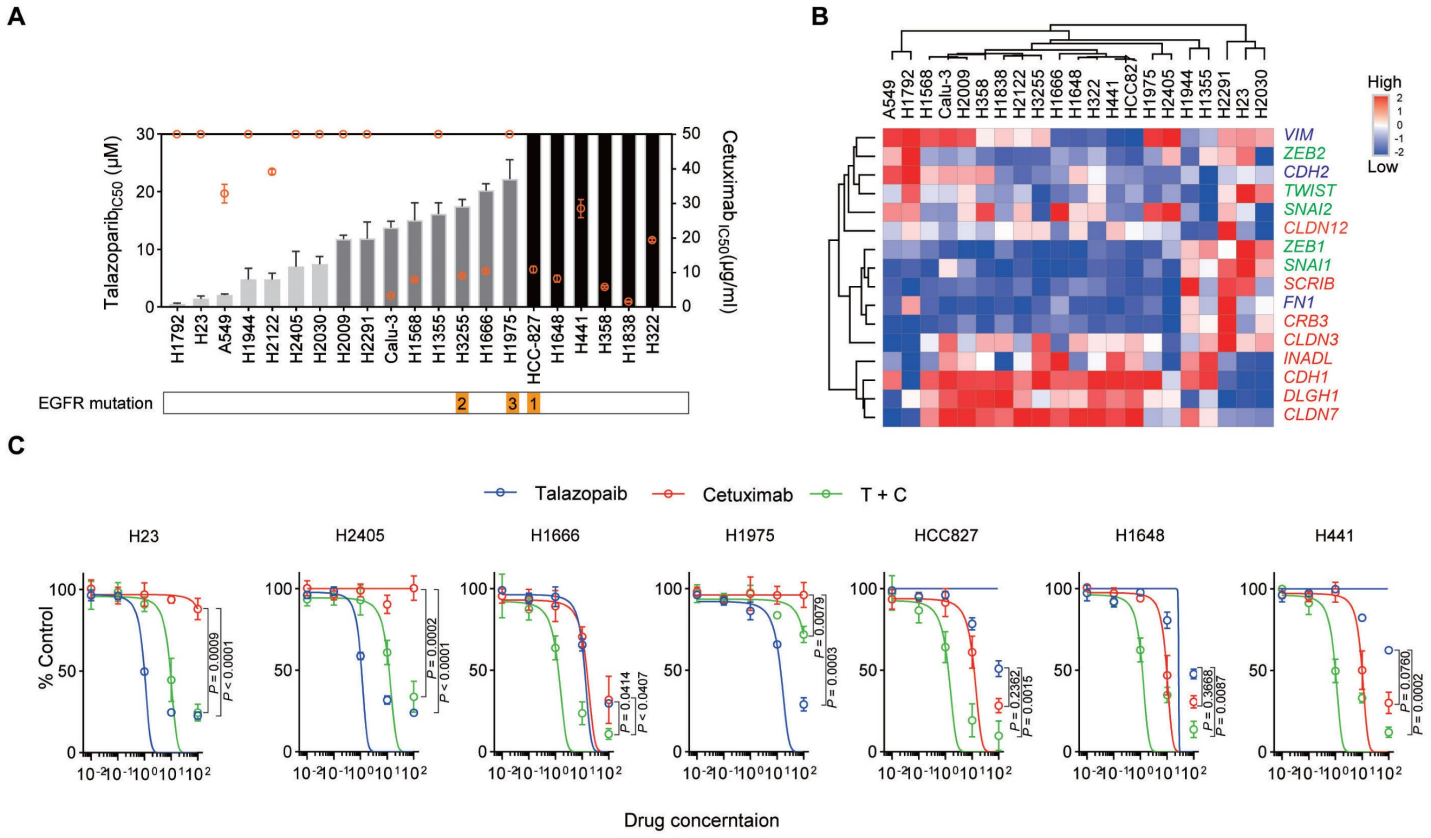
** Cetuximab and talazoparib have a synergetic effect in NSCLC cells**. **A.** Inhibition of cell growth induced by talazoparib (left y-axis, data presented in columns) and cetuximab (right y-axis, data presented in a plot) was determined in a panel of NSCLC cell lines and is reported as the IC50 assessed from dose‒response curves from a minimum of three experiments. NSCLC cell lines exhibiting a talazoparib IC_50_ < 10 μM, highly responsive; IC_50_ =10 μM to 30 μM, moderately responsive; IC_50_ > 30 μM, unresponsive cells, while for cetuximab, IC_50_ < 20 μg/ml, highly responsive; IC_50_ =20 to 50 μg/ml, moderately responsive; IC_50_ > 50 μg/ml, unresponsive cells. **B.** Heatmap representing the transcript expression of select markers, including vimentin and N-cadherin (VIM and CDH2), FN1, zinc finger E-box binding homeobox 1 (ZEB1), ZEB2, TWIST, SNAI1, SNAI2, E-cadherin (CDH1), INADL, SCRIB, CRB3, DLGH1, and claudins, as determined by quantitative polymerase chain reaction (qPCR) analysis. Red, epithelial-associated genes; green, EMT transcription factors; blue, mesenchymal differentiation markers. **C.** NSCLC cells were treated with increasing concentrations of the indicated drugs [talazoparib (μM), cetuximab (μg/ml)]. Cell proliferation relative to an untreated control was measured after 4 days using alamarBlue staining. Data are presented as the mean ± s.d. of six independent biological replicates. *P* values were obtained using two-way ANOVA followed by a Bonferroni post-test.

The median inhibitory concentration (IC50) values of talazoparib and cetuximab were tested in a panel of NSCLC cell lines (adenocarcinoma cells) (Figure 1A and Table S1). Interestingly, responses to the two drugs were not consistent across the cell lines used in our study. We also assessed the expression of EMT markers in these NSCLC cell lines (Figure 1B), supporting the potential correlation between cetuximab activity and the EMT process, as previously reported. However, EMT markers were not correlated with talazoparib activity. We chose some cell lines for further combined treatment study: (1) H23 and H2405 (highly talazoparib-responsive but cetuximab-unresponsive cells); (2) H1666 (moderately talazoparib-responsive and cetuximab-responsive cells); (3) HCC827 and H1648 (highly cetuximab-responsive but talazoparib-unresponsive cells); and (4) H441 (moderately cetuximab-responsive and talazoparib-unresponsive cells). These cell lines were treated with monotherapy or cetuximab plus talazoparib over a wide range of drug concentrations (Figure 1C). Our data showed that for H23, H2405, and H1975 cells, talazoparib plus cetuximab treatment did not yield a stronger inhibitory effect than monotherapy (talazoparib treatment alone), while for H1666, HCC827, H1648 and H441 cells, combined therapy with talazoparib plus cetuximab dramatically improved the inhibitory effect. Notably, H1666, HCC827, H1648 and H441 cells were all responsive to cetuximab and expressed enriched epithelial-like markers, while H23, H2405, and H1975 cells were not responsive to cetuximab and expressed enriched mesenchymal markers.

The dynamics of stem cell-like properties are impacted by cetuximab and talazoparib. Next, we investigated whether talazoparib or cetuximab in combination with talazoparib therapy might affect the proportion and quantity of cancer stem cells (CSCs) in NSCLC cell lines. Based on the IC50 values for various NSCLC cell lines discovered in cytotoxicity assays, the treatment dose was chosen. Aldehyde dehydrogenase (ALDH) activity and cell surface CD133 expression were both used as stem cell markers for NSCLC; as previously reported, the tumorigenicity of different cell populations derived from NSCLC cell lines in vivo was evaluated using the following markers: (1) CD133^+^ALDH^-^ cells (enriched mesenchymal-like CSCs); (2) CD133^-^ALDH^+^ cells (enriched epithelial-like CSCs); (3) CD133^+^ALDH^+^ cells (epithelial-to-mesenchymal transition-like CSCs); and (4) CD133^-^ALDH^-^ cells (differentiated tumour cells, bulk tumour cells). We validated the tumorigenic capacity of cell populations obtained from HCC827, H1648, H1666, H441, and H2405 because the definition of cancer-initiating cells rests mostly on functional features. Consistent with earlier results 37, only the CD133^-^ALDH^-^ population failed to repopulate the tumour (Table 1).

**Figure 2:**
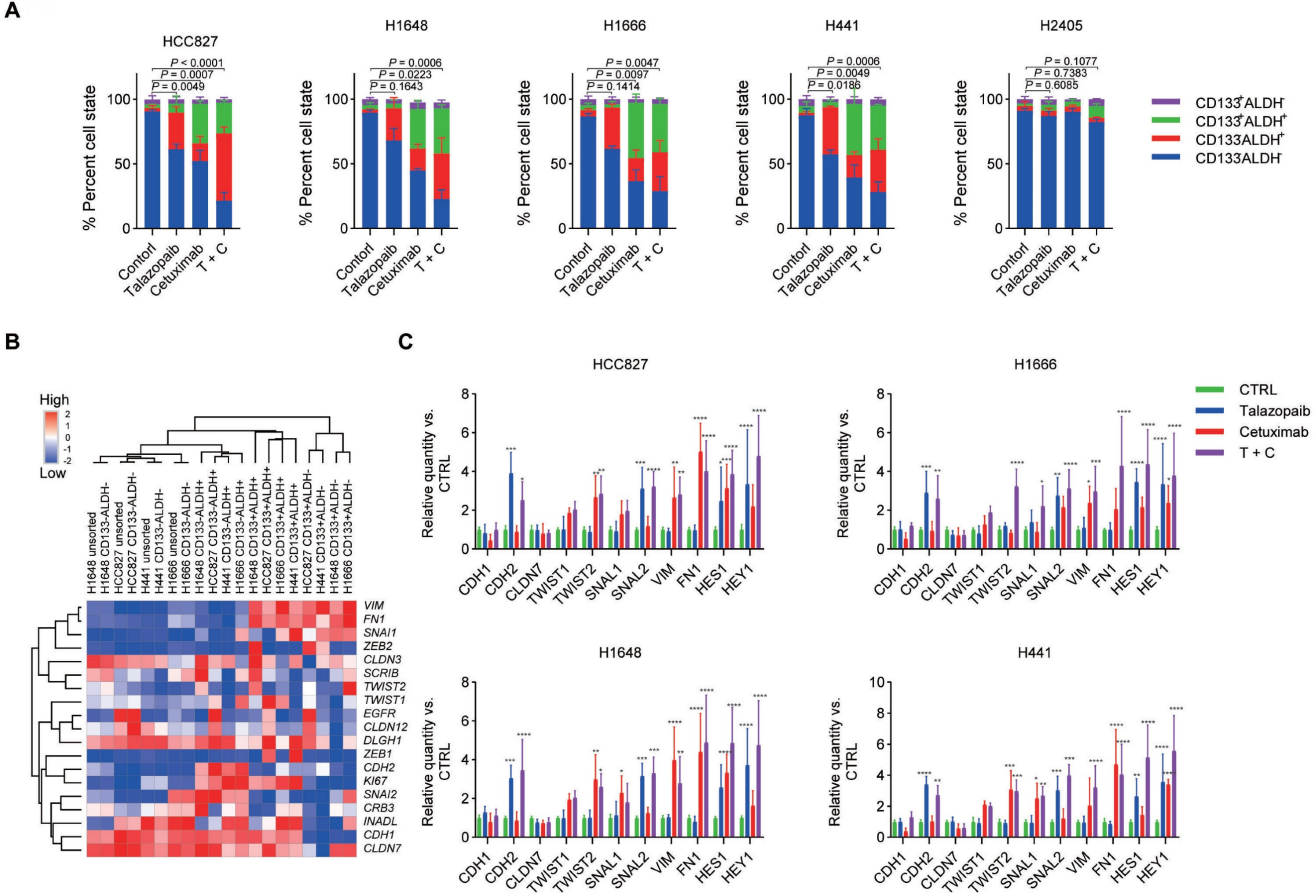
** EGFR and PARP blockade increases the CSC subpopulation of NSCLC cells. A.** NSCLC cells were treated with cetuximab, talazoparib, or cetuximab plus talazoparib for 4 days, and the resulting cells were analysed by fluorescence-activated cell sorting (FACS). Each drug was applied at a concentration that resulted in 50% inhibition of viability, and combined therapy was applied at half the dose of each drug. For H2405 cells, cetuximab was applied at 50 μg/ml. **B.** Heatmap representing the expression of selected transcripts in different cell subpopulations, as determined by qPCR analysis.** C.** NSCLC cells were treated with different drugs on Day 1. Select gene expression was determined by qPCR analysis after 72 hours. Gene expression was normalized to that of the housekeeping gene β-actin and is expressed as the fold difference relative to that of the control cells. Data are presented as the mean ± s.d. of six independent biological replicates in A and C. *P* values were obtained using one-way ANOVA followed by Tukey's post-test (A, percentage of ALDH+ cells) or two-way ANOVA followed by Bonferroni's post-test (C). *, *P* < 0.05; **, *P* <0.01; ***, *P* < 0.001; ****, *P* < 0.0001.

The results showed that both EGFR inhibition and talazoparib treatment affect the dynamics of the stem cell-like properties of ALDH^+^ cells but not ALDH^‑^ cells. In particular, talazoparib treatment significantly increased the frequency of CD133^-^ALDH^+^ cells, while cetuximab treatment increased both CD133^-^ALDH^+^ cells and CD133^+^ALDH^+^ cells (the fraction of CD133^+^ALDH^+^ cells was higher than that of CD133^-^ALDH^+^ cells) (Figure 2A and Figure S1). Moreover, combined therapy with cetuximab plus talazoparib yielded a stronger enrichment of both the CD133^+^ALDH^+^ cell and CD133^-^ALDH^+^ cell subpopulations. Interestingly, we did not observe a significant change in the dynamics of stem cell-like properties in the H2405 cells after treatment, suggesting a different mechanism in the cells expressing high levels of mesenchymal markers.

Due to the strong correlation between the EMT process and CSCs 38, in isolated cell subsets, we further examined the expression levels of mesenchymal- and epithelial-associated genes. According to our findings, the CD133^-^ALDH^+^ cell subsets expressed more epithelial-related genes than other cell subsets (Figure 2B). Additionally, the CD133+ALDH- subpopulations upregulated alternative EMT transcription factors and mesenchymal differentiation markers, while the bulk cells maintained a gene expression pattern resembling that of their parental cells. In addition, we found that the CD133^+^ALDH^+^ subpopulations expressed both mesenchymal- and epithelial-like markers, as previously reported 39. Consistent with the enrichment of CD133^+^ALDH^+^ stem-like subpopulations, several NSCLC cell lines, including H1666, HCC827, H1648 and H441 cells, showed unchanged expression of epithelial-associated genes. While the expression of mesenchymal-associated genes was upregulated after cetuximab treatment, talazoparib treatment did not significantly interfere with the expression of mesenchymal-associated genes (Figure 2C). Notably, the expression of the *CDH2* gene was upregulated after talazoparib but not cetuximab treatment in all tested cell lines, which is consistent with the fact that CD133^-^ALDH^+^ stem-like subsets eventually express high levels of *CDH2*. Although treatment with cetuximab plus talazoparib showed a strong synergistic antiproliferative effect in the in vitro assay, this treatment significantly upregulated the expression of mesenchymal-associated genes. The protein expression of EMT makers CDH1 and VIM in H1648 and HCC827 cells were further confirmed by protein array (Figure S2) and immunofluorescence assays (Figure S3).

### Notch receptors play crucial roles in the enrichment of stem-like cells

**Figure 3:**
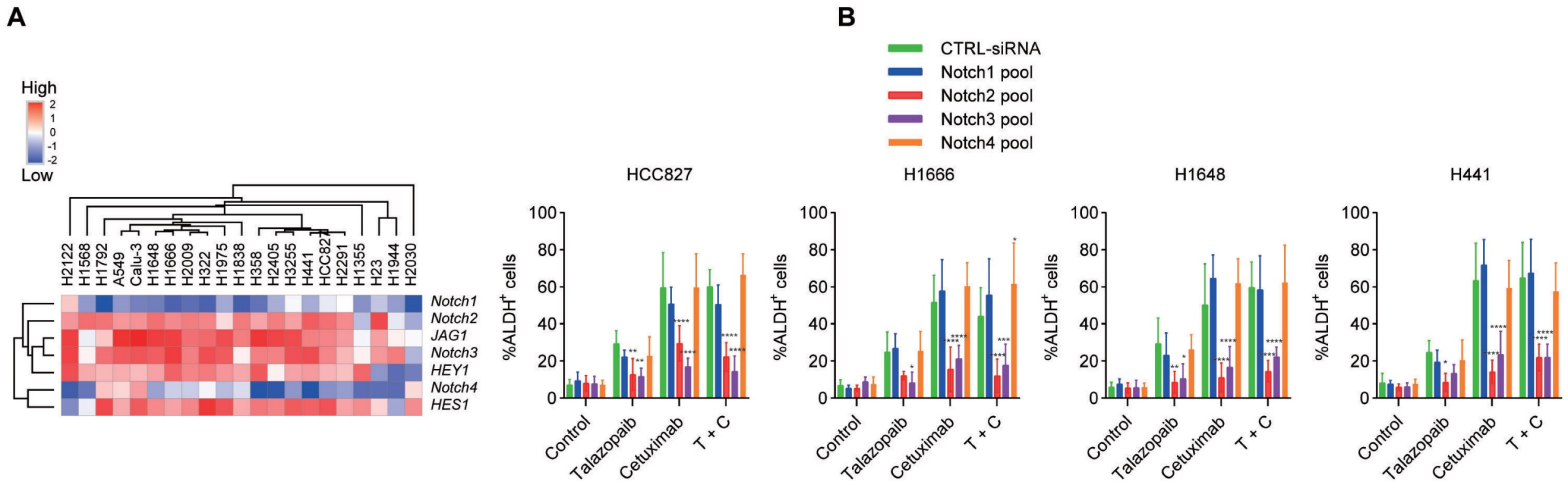
** Notch signalling is crucial for EGFR/PARP blockade-induced CSC enrichment. A.** Heatmap representing the transcript expression of Notch signalling genes, as determined by qPCR analysis. **B** Cancer cells were transfected with a CTRL pool (CTRL-siRNA) and a pool of Notch siRNAs. After 48 hours, the cells were treated as specified in the figure for 3 days, and ALDH activity was measured. Data are presented as the mean ± s.d. of six independent biological replicates. *P* values were obtained using two-way ANOVA followed by a Bonferroni post-test. *, *P* < 0.05; **, *P* <0.01; ***, *P* < 0.001; ****, *P* < 0.0001; versus the CTRL.

Since our research 32, 33 and that of others 12 has shown that Notch signalling is essential for the growth of ALDH+ cells in NSCLC, we next examined the function of Notch signalling in the enrichment of cancer stem-like cells caused by EGFRi and PARPi. H1648, Calu-3, H1666, H2122, A549, H1975, and H2405 cells exhibited the highest expression of Jagged1 among the cancer cell lines used (Figure 3A). Additionally, Notch2 and Notch3 had higher expression levels than Notch1 and Notch4, which is consistent with a previous study 40. The target genes of Notch signalling, *HES1* and *HEY1*, showed noticeably increased expression following treatment with cetuximab or talazoparib (Figure 2C). Moreover, the expression of the *HES1* and *HEY1* genes increased after combined treatment with both cetuximab and talazoparib. Drug-induced ALDH^+^ cell expansion in H1666, HCC827, H1648 and H441 cells was dramatically inhibited after treatment with Notch2 and Notch3 siRNAs, whereas those treated with Notch1 and Notch4 siRNAs showed negligible changes (Figure 3B).

### EGFR/Notch bsAb enhances the sensitivity of cancer cells to talazoparib

**Figure 4:**
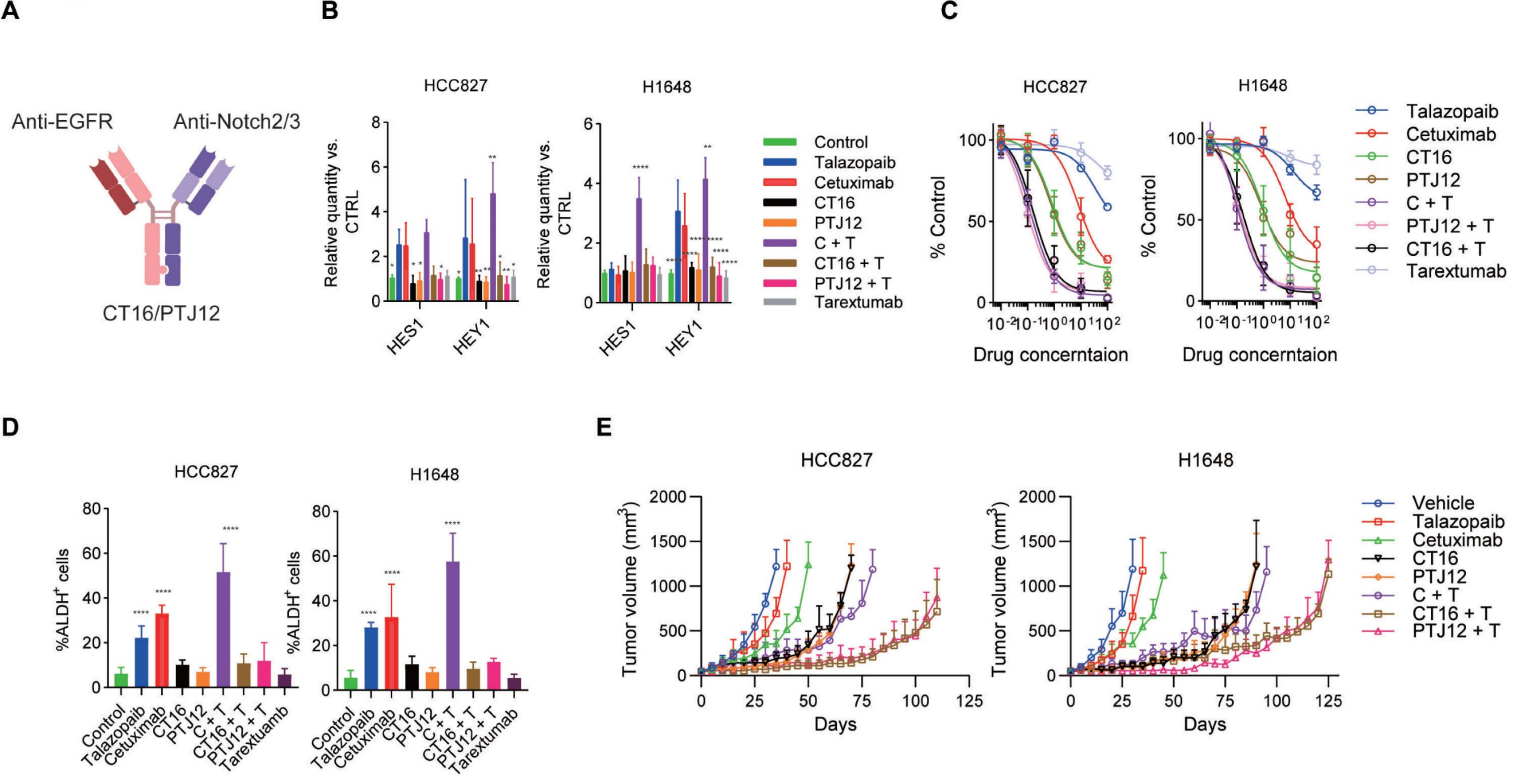
** Blockade of EGFR, PARP and Notch effectively prevents resistance. A**, Schematic representation of CrossMab CT16 and PTJ12. **B**, Cells were treated with the drugs specified in the figure, and the expression of selected genes was determined by qPCR analysis. Gene expression was normalized to the housekeeping gene β-actin and is expressed as the fold change compared to that of the control cells. **C**, NSCLC cells were treated with increasing concentrations of the indicated drugs [talazoparib (μM), antibodies (μg/ml)]. Cell proliferation relative to an untreated control was measured after 4 days using alamarBlue staining. **D**, NSCLC cells were treated with different drugs for 4 days, and the resulting cells were analysed via FACS. **E**, Tumour-bearing mice were treated weekly with cetuximab (30 mg/kg), talazoparib (100 mg/kg), CT16 (30 mg/kg), or PTJ12 (30 mg/kg) alone or in combination with antibodies plus talazoparib (15 mg/kg + 50 mg/kg), as specified in the figure. n = 8 per group. Data are presented as the mean ± s.d. of six independent biological replicates (B-D). *P* values were obtained using two-way ANOVA followed by a Bonferroni post-test (B, C) or one-way ANOVA followed by a Tukey post-test (d). *, *P* < 0.05; **, *P* <0.01; ***, *P* < 0.001; ****, *P* < 0.0001; versus the CTRL.

The above data strongly confirm the efficacy of a combination of EGFR inhibitors, PARP inhibitors, and Notch signalling blockers. Previously, two bispecific antibodies, CT16 and PTJ12 (Figure 4A), were generated using the "knobs into holes" and CrossMab methodologies. These bispecific antibodies have a high affinity for EGFR and Notch2/3, potently inhibiting ligand binding-induced phosphorylation of EGFR and downstream signalling and inhibiting ligand binding to human Notch2 and Notch3 receptors, effectively impairing their reporter activity. Therefore, we used these bispecific antibodies in this study based on their established function as potent inhibitors of ligand binding and signalling activation.

We evaluated the inhibitory efficacy of the bispecific antibodies against drug treatment-induced Notch activation. Dual blockade of EGFR and Notch signalling with bispecific antibodies or combined treatment with bispecific antibodies plus talazoparib significantly inhibited drug-induced activation of the *HES1* and *HEY1* genes, which are downstream Notch signalling molecules in HCC827 and H1648 cells (Figure 4B). The effect of these antibodies to EGFR and Notch signalling were further determined by protein array (Figure S4). Similar results were obtained in H1666 and H441 cells (Figure S5a). In cell proliferation assays, a dose-dependent reduction in proliferation was observed after treatment with the EGFR-targeting antibody cetuximab and the PARP inhibitor talazoparib. Interestingly, significant inhibition of cell proliferation occurred after treatment with cetuximab plus talazoparib, and this effect was even stronger than the antitumour effect of bispecific antibodies. Combined therapy with bispecific antibodies plus talazoparib led to a dramatic inhibition of cell growth (Figure 4C and Figure S5B) and showed a strong effect on cell apoptosis (Figure S5C). Notably, in this in vitro assay, bispecific antibodies plus talazoparib did not show better antitumour efficacy than cetuximab plus talazoparib treatment. Next, we sought to confirm the anti-stem cell effect of these bispecific antibodies plus talazoparib. In both HCC827 and H1648 cell lines, the combination of bispecific antibodies and talazoparib showed a considerable inhibition of the enrichment of the ALDH^+^ subsets (Figure 4B). We also tested the anti-stem cell effect of bispecific antibodies together with talazoparib in H1666 and H441 cell lines (Figure S5D) and obtained similar results.

To assess the potential effectiveness of this multitargeting regimen for NSCLC, we next compared the antitumour effects of bispecific antibodies combined with talazoparib in mice xenografted with either H1648 or HCC827 cells. A dose of 30 mg/kg was selected for the bispecific antibodies, as was previously reported. Talazoparib was given at a dose of 100 mg/kg because, in our toxicity study, NOD-SCID (nonobese diabetic-severe combined immunodeficient) mice and BALB/c nude mice both experienced significant weight loss at a dose of 125 mg/kg. As shown in Figure 4E, treatment with the bispecific antibodies CT16 and PTJ12 significantly slowed tumour growth to this volume for approximately 35 days. In contrast, the vehicle-treated HCC827 tumours advanced quickly, reaching a volume of 1,000 mm^3^ in less than 30 days. Both CT16 and PTJ12 treatments effectively slowed tumour growth in H1648 tumours for a period of approximately 60 days. Moreover, cetuximab plus talazoparib treatment showed antitumour benefits similar to those of bispecific antibodies. However, single cetuximab treatment only had a modest therapeutic effect, whereas single talazoparib treatment did not exhibit a marked inhibition of tumour growth in either tumour model. As acquired resistance ultimately occurs after prolonged exposure of cancer cell lines to specific drugs, a process in which an increase in stem-like cancer cells has been implicated, next, we investigated whether coblockade of EGFR/PARP activity and Notch signalling would postpone or stop the development of drug resistance in vivo. Notably, as shown in Figure. 4E, although a significant delay in tumour growth was observed in the HCC827 tumours treated with cetuximab plus talazoparib for almost 40 days and in the H1648 tumours treated for approximately 50 days, the tumours subsequently progressed rapidly. This result suggests the development of variants with acquired resistance to both EGFR and PARP inhibitors, consistent with the demonstration that these cells can regrow in the presence of both cetuximab and talazoparib. Notably, treatment targeting EGFR, PARP and Notch using bispecific antibodies and talazoparib delayed the rapid development of resistance for an additional 40 to 50 days, suggesting significantly enhanced efficacy of the triple-targeting regimen. Interestingly, although cetuximab plus talazoparib treatment caused a notable increase in the fraction of stem cell-like cells, this combined treatment induced a strong initial response of monolayer cultured cells in the in vitro assays, with 25% and 9% inhibition of cell proliferation. The antitumour efficiency of cetuximab plus talazoparib in primary tumours was further verified using in vivo models of HCC827 and H1648 tumours, further supporting the therapeutic value of this combination. Blockade of EGFR and Notch signalling with bispecific antibodies or combined treatment with bispecific antibodies plus talazoparib significantly inhibited drug-induced activation of the Notch2 and Notch3 cleavage (Figure S6A), and also significant decrease the fraction of ALDH^+^ cells (Figure S6B) in the in vivo treatment of HCC827 and H1648 tumours.

We further evaluated the blockade effect of bispecific antibodies plus talazoparib on tumour resistance in the established resistant tumour models. Cancer cells that underwent EGFR/PARPi treatment or control cells were retransplanted into a new cohort of mice, and defined numbers of cancer cells were observed for the assessment of tumour growth inhibition in the presence of different antibodies. HCC827 and H1648 cells with resistance to both cetuximab and talazoparib were termed 827C, 827T, 1648C, and 1648T cells. Our data suggested that cancer cells also lost sensitivity to other types of inhibitors if they obtained resistance to one inhibitor, and bispecific antibodies showed little benefit in tumour growth compared to monotherapy in such situations (Table S2).

### Combined therapy with EGFR/Notch bsAb and PARPi decreases CSC frequency and delays tumour recurrence after radiation therapy

**Figure 5:**
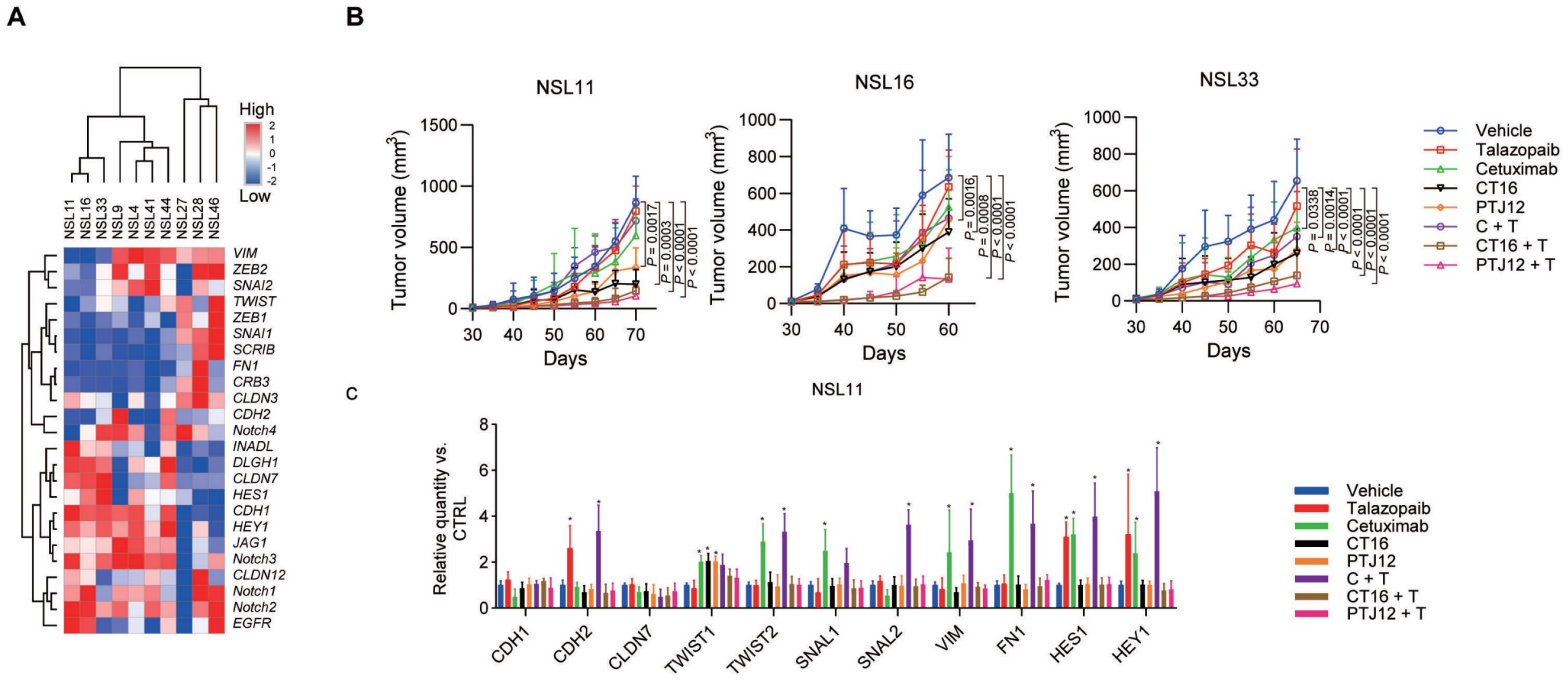
** Combined therapy with bispecific antibodies plus talazoparib is effective, as multiple therapies alter tumour cell EMT status. A**. Heatmap representing the expression of selected transcripts in different tumour samples, as determined by qPCR analysis. **B**. Different treatments inhibited tumour growth in NSCLC models. Tumour-bearing mice were treated weekly with cetuximab (30 mg/kg), talazoparib (100 mg/kg), CT16 (30 mg/kg), or PTJ12 (30 mg/kg) alone or in combination with antibodies plus talazoparib (15 mg/kg + 50 mg/kg), as specified in the figure. n = 8 per group. **C**. qPCR analysis was conducted to determine the expression of selected genes in NSL11 tumours subjected to different treatments. Gene expression was normalized to that of the housekeeping gene β-actin and is expressed as the fold change compared with the vehicle group. Data are presented as the mean ± s.d. (B) or mean ± s.d. of six independent biological replicates (C). *P* values were obtained using two-way ANOVA followed by a Bonferroni post-test (B-C); *, *P* < 0.05 versus the CTRL.

We next constructed patient-derived tumour xenograft (PDX) models in NOD-SCID mice with minimally passaged human tumours to further explore the antitumour effects of bispecific antibodies combined with talazoparib. Engraftment and drug responses in these mouse models were associated with clinical outcomes and patient responses after treatment. In Supplementary Table S2, the histologic characteristics of the tumours that have been treated are compiled. Human EGFR, Notch1, Notch2, and Notch3 mRNA were all expressed in tumours (Figure 5A). A prophylactic dosing regimen was performed in the initial xenograft studies, and initial treatment with different inhibitors was applied two days after the implantation of tumour cells. Our data showed a decrease in tumour growth in 3 of the 10 NSCLC tumour xenografts after cetuximab treatment, whereas 7 of the 10 xenografts were responsive to talazoparib (Table S3). Cetuximab plus talazoparib treatment elicited a significantly notable response in the monolayer cultured cells in vitro and the xenograft models in vivo, supporting a synergistic effect of these two agents, and combination therapy showed more potent antitumour activity than cetuximab treatment in the cetuximab-sensitive NSCLC PDX models, which suggests a specific role of PARP-EGFR cross-talk in PDX models (Figure 5B and Table S4). Our data also showed that the combination of bispecific antibodies plus talazoparib, but not a bispecific antibody alone, was particularly effective for suppressing tumour growth compared with monotherapy, combined treatment with cetuximab and talazoparib or individual treatment with bispecific antibodies in PDX models with sensitivity to EGFR blockade. The antitumour efficacy of bispecific antibodies plus talazoparib was not improved in the tumours that were insensitive to EGFR blockade, corresponding to the high expression of *VIM* and *FN1* genes and relatively low expression of *CDH1* and *CLDN7* genes (Figure 5C). In addition, the results of an immunohistochemical study of NSL11 tumour showed that cetuximab or talazoparib resulted in a significant increase in the expression of VIM and a significant decrease in the expression of CDH1. On the other hand, treatment with either bispecific antibodies or bsAb plus talazoparib significantly reduced VIM expression in the tumor cells. In previous reports 41, EMT markers have been reported to be useful in predicting the response to EGFR inhibitors. Our data suggested that EMT markers are also associated with sensitivity to the synergistic effect of bispecific antibodies plus talazoparib, suggesting that they are valuable markers in NSCLC. The upregulation of *VIM* and *FN1* gene expression induced by EGFR blockade and upregulation of CDH2 gene expression induced by talazoparib (Figure 5C and Figure S7) were significantly blocked by CT16 or PTJ12 (P <0.0001).

**Figure 6:**
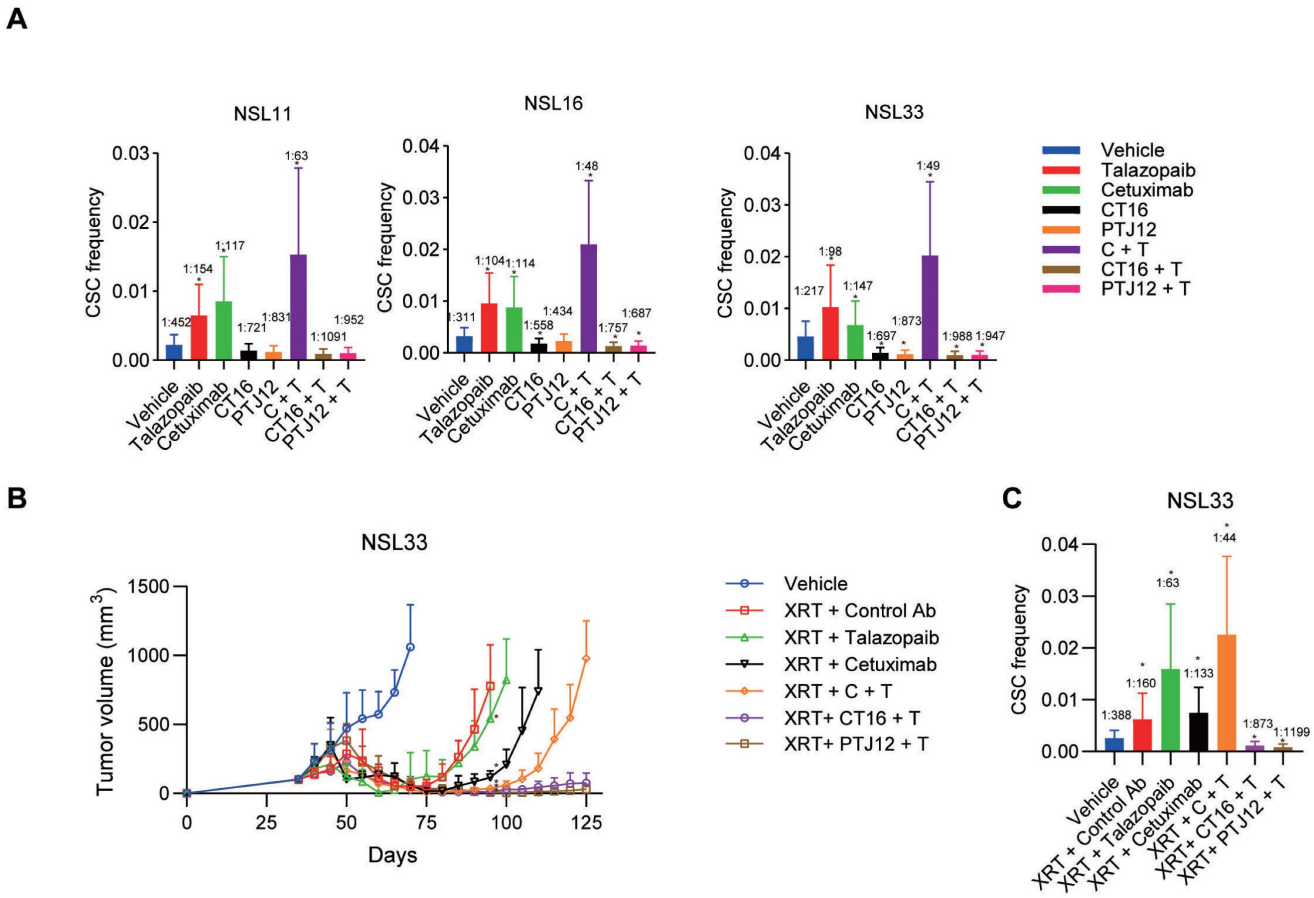
** Combined treatment decreases the CSC frequency and has superior antitumour activity when combined with radiation. A,** Limiting dilution analysis of the ability of cells isolated from treated NSCLC tumours to initiate tumour growth in the absence of further treatment after secondary transplantation into a new cohort of mice. The primary tumour was subjected to different treatments as indicated. The CSC frequency refers to the average number of cells required to cause tumour growth in the recipient cohort.** B,** The effect of different drugs [weekly treatment; control IgG (30 mg/kg), cetuximab (30 mg/kg), talazoparib (100 mg/kg), CT16 (30 mg/kg), PTJ12 (30 mg/kg), and combined therapy with cetuximab/talazoparib, CT16/talazoparib, or PTJ12/talazoparib (15 mg/kg + 50 mg/kg)] on NSCLC NSL33 tumour recurrence after discontinuation of XRT (weekly 10 Gy for 3 weeks). n = 8 per group. **C,** The effect of different treatments on CSC frequency in NSL33 tumours at the end of the in vivo study. Data are presented as the mean ± s.d. (B) or mean ± upper/lower limits (A and C). *P* values were obtained using two-way ANOVA followed by a Bonferroni post-test at Day 95 (b) or using one-way ANOVA followed by a Tukey post-test (A-C); *, *P* < 0.05 versus the CTRL Ab (B) or vehicle.

Limiting dilution studies were conducted to evaluate the impact of different treatment regimens on CSC (TIC) frequency. Defined numbers of human tumour cells derived from different treatment groups or vehicle controls were retransplanted into a new cohort of mice without further treatment to assess their ability to initiate tumour growth (Figure 6A). PDXs treated with talazoparib exhibited a 2.2- to 3.0-fold increase in TIC frequency among the tumour cells. Neither cetuximab nor talazoparib treatment of NSCLC reduced the tumorigenic cell frequency. EGFR/PARP inhibitors actually increased tumorigenicity, while combined therapy with cetuximab plus talazoparib strongly enhanced tumorigenicity. In contrast, both CT16 and PTJ12 reduced tumorigenicity by 1.5- to 4.0-fold in all tumours. Of note, when talazoparib was added, treatment with bispecific antibodies plus talazoparib led to a notable 2.4- to 4.5-fold reduction in tumorigenicity in all tumours.

Since the combination of PARP inhibitors and radiotherapy may be a promising regimen to locally enhance DNA damage in tumour cells, we further assessed the effect of triple-targeting agents on tumour recurrence after radiotherapy. In our study, NSCLC tumours were treated with a high dose of radiation (10 Gy ×3), leading to sufficient tumour regression. However, the surviving tumour cells began to grow into large tumours four weeks after the cessation of XRT, and the CSC subpopulation was enriched in the residual tumour cells after irradiation, with an approximate 2-fold increase in CSC frequency compared to the vehicle group (Figure 6B-C). However, although the combination of cetuximab and XRT led to almost complete tumour regression, the residual cancer cells were capable of regrowing after monotherapy with cetuximab. Moreover, after the combined treatment, the surviving tumour cells exhibited a significant increase in CSC frequency, which was approximately 2-fold greater than that in the group treated with XRT only. In addition, the regimen of talazoparib plus cetuximab delayed the occurrence of tumour regrowth by 9 weeks after the cessation of XRT, reducing the CSC frequency to 30% relative to that of the XRT group and 60% relative to that of the XRT + cetuximab group. The therapeutic efficacy of the bispecific antibodies plus talazoparib persisted after termination of XRT and antibody treatments. The triple-targeting treatment reduced the proportion of CSCs in the surviving tumour cells and the tumourigenicity, with a 5-fold and 10-fold decrease in CSC frequency compared to that in the vehicle group and the single XRT treatment group, respectively. Together, these results demonstrate the efficacy of the EGFR, Notch and PARP triple-targeting strategy for the treatment of NSCLC tumours, resulting in inhibition of bulk tumour cell growth and reduced tumorigenic cell frequency.

### Antitumour effect of combination therapy in pancreatic, ovarian, and breast cancer

We assessed the antitumour efficacy of triple-targeting therapy in a number of additional xenografts, such as pancreatic, ovarian, and triple-negative breast (TNBC) tumours, in addition to NSCLC tumours. In PN21 (a pancreatic tumour), ON33 (a serous ovarian tumour), and BN16 (a TNBC tumour), EGFR/Notch-targeting bispecific antibodies demonstrated significant efficacy in combination with the PARP inhibitor talazoparib (Figure S8A). Notably, in PN21 tumours, PTJ12 plus talazoparib treatment reduced the CSC frequency to 80% compared to that in the control group, whereas enriched CSCs were found among the residual tumour cells after talazoparib treatment, with an approximately 1.5-fold increase in CSC frequency compared to that in the control group (Figure S8B). Notably, talazoparib and bispecific antibodies together reduced tumorigenicity and the frequency of CSCs in residual tumour cells, with a 7-fold decrease in CSC frequency compared to that of the group receiving talazoparib alone. ON33 and BN16 xenografts showed comparable outcomes (Figure S8B). Overall, the results show that the EGFR/Notch/PARP triple-targeting strategy has promise for the treatment of a variety of solid tumours.

## Discussion

A large family of proteins known as poly (ADP-ribose) polymerase (PARP) is involved in numerous cytoplasmic and nuclear activities in cells 42. The most prevalent of these, PARP-1, is a poly-ADP-ribosylating (PARylating) chromatin-associated enzyme that plays a role in DNA damage repair, transcriptional control, genomic stability, cell transformation, and cell death 43, 44. Since its discovery, most studies have examined the role of PARP-1 in sensing and repairing DNA damage 45. PARP-1 was found to bind to damaged DNA strands through N-terminal zinc-finger motifs, followed by activation of the C-terminal catalytic domain, which then hydrolyses NAD+ and generates poly ADP-ribose (PAR) chains 46. Nonetheless, interest in the role of PARP-1 in gene regulation has substantially grown over the past decade 47, 48. PARP-1 was also reported to contribute to the regulation of mitochondrial content and metabolism and to the production of reactive oxygen species (ROS) through its control over NAD^+^ levels and some key metabolism-related transcriptional regulators, including NRF2^49^. PARP inhibitors have been shown to have specific efficacy against tumours with BRCA1/2 mutations, leading to the subsequent success of the use of PARP inhibitors in the treatment of certain tumours with BRCA mutation and HR deficiency 50. This advance helped with the development of other therapeutic agents that target key nodes in the DDR network, including ATM. Moreover, the search for specific mutations, the presence of which determines the activity of certain drugs, and the exploration of mechanistic combination therapies are expected to enhance the activity of these drugs 50. However, many tumours lack certain mutations that determine drug sensitivity. Furthermore, most clinically advanced therapies using these sensitivity-dependent agents in combination involve chemotherapeutics that lead to DNA damage, inhibitors targeting other nodes in the DDR network, or chromosomal modifiers. However, these combined strategies may cause increased toxicity not only to tumour cells but also to normal cells. Clinical trials using PARP inhibitors combined with chemotherapeutics, such as cisplatin, gemcitabine, and temozolomide, have shown exacerbated toxicity, with the need to decrease the therapeutic dose, indicating a narrow therapeutic index 50.

Numerous studies have revealed that activation of EGFR signalling induces multiple cellular stress responses, including increased oxidative stress, proliferation, and replication, all of which lead to DNA damage 51. Therefore, an enhanced capacity for DNA damage repair is required for genomic stability, as EGFR activation provides vulnerability to therapies targeting DNA damage repair. Notably, the present study strongly suggests the use of EGFR-targeting antibodies to potentiate PARP inhibitor effects: (1) cetuximab and talazoparib showed synergistic effects both in vitro and in vivo; (2) cetuximab and talazoparib both influenced the dynamics of stem cell-like properties but showed enrichment of different cell subtypes; and (3) enrichment of cell subpopulations highly expressing mesenchymal markers was observed under treatment with EGFR inhibitors but not talazoparib. Therefore, we demonstrate that targeting oncogenic genes such as EGFR in cancer cells treated with PARP inhibitors may potentially improve the therapeutic depth and duration of PARP-targeting therapies, which also fits into the exploration of targets in the DNA damage response (DDR) network for potential cancer therapeutic strategies. Previous studies have demonstrated a synergistic killing effect of combined EGFR and PARP blockade in cancer cells 52, and our data also suggest that combination therapy with EGFR and PARP inhibitors has the potential to be an effective therapeutic strategy for multiple tumours beyond hereditary BRCA1- and BRCA2-deficient tumours. Notably, in this report, we use both EGFR wild type and EGFR mutant cancer cell lines. Although EGFR mutation which might result in the activation of ligand-independent tyrosine kinases, these cell lines may aslo respond to cetuximab EGFR in our results and previous reports, as EGFR targeting antibody has several mechanisms: to block ligand-dependent EGFR signalling, to induce EGFR degradation, and block EGFR-ERBB interaction, et al. 53.

Interestingly, the present evidence suggests that PARP or EGFR blockade affects the enrichment and relative proportion of ALDH^+^ cells among tumour cells. Moreover, in PDX models, dual targeting of EGFR and PAPR further increased the CSC frequency. Our data reveal that the combination of CSC-targeting agents and those targeting DDR-related molecules has translational potential for the treatment of various malignancies based on the broad application of these therapeutic strategies in clinical practice. Moreover, the study proposed a specific mechanism of Notch signalling inhibition that prevents acquired resistance not only to individual EGFR inhibitors but also to PARP blockers.

## Conclusion

Our results initially uncovered the significance of the combined therapy of cetuximab plus talazoparib in promoting the eventual enrichment of stem-like cells and EMT, although the pharmacological combination of cetuximab and talazoparib showed a strong antitumour effect in vitro. We suggest that coblockade of EGFR and Notch signalling can effectively suppress both bulk tumour cells and CSCs, which also promotes the response after PARP blockade in different cancer cell lines and PDX tumour models. Our results support the notion that PARP inhibitors administered in combination with immunotherapy using EGFR/Notch bsAbs have broad potential for the treatment of various tumours beyond hereditary BRCA1-deficient and BRCA2-deficient tumours, including lung cancer.

## Supplementary Material

Supplementary figures and tables.

## Figures and Tables

**Table 1:** In vivo Tumor Development Experiments in NOD/SCID Mice

		Tumors detected/injections*		
		100000	10000	1000
HCC827	CD133^+^ALDH^-^	5/5	4/5	4/5
	CD133^+^ALDH^+^	—	5/5	3/5
	CD133^-^ALDH^-^	1/5	0/5	0/5
	Unsorted	5/5	4/5	3/5
H1648	CD133^+^ALDH^-^	—	5/5	3/5
	CD133^+^ALDH^+^	—	5/5	3/5
	CD133^-^ALDH^-^	0/5	0/5	0/5
	Unsorted	5/5	4/5	4/5
H1666	CD133^+^ALDH^-^	5/5	4/5	3/5
	CD133^+^ALDH^+^	—	5/5	4/5
	CD133^-^ALDH^-^	0/5	0/5	0/5
	Unsorted	5/5	4/5	4/5
H441	CD133^+^ALDH^-^	5/5	4/5	4/5
	CD133^+^ALDH^+^	5/5	43/5	2/5
	CD133^-^ALDH^-^	0/5	0/5	0/5
	Unsorted	5/5	4/5	3/5
H2405	CD133^+^ALDH^-^	4/5	3/5	3/5
	CD133^+^ALDH^+^	5/5	4/5	3/5
	CD133^-^ALDH^-^	0/5	0/5	0/5
	Unsorted	5/5	4/5	4/5

*The number of tumors detected divided by the number of injections in mice (*N* = 5).

## References

[1] Arteaga CL, Engelman JA (2014). ERBB receptors: from oncogene discovery to basic science to mechanism-based cancer therapeutics. Cancer Cell.

[2] Moscatello DK, Holgado-Madruga M, Godwin AK, Ramirez G, Gunn G, Zoltick PW (1995). Frequent expression of a mutant epidermal growth factor receptor in multiple human tumors. Cancer Res.

[3] Devarakonda S, Morgensztern D, Govindan R (2015). Genomic alterations in lung adenocarcinoma. Lancet Oncol.

[4] Li T, Fu W, Lei C, Hu SJNSAfTA-EA Current status of anti-EGFR agents. 2023: 1-12.

[5] Ma Z, Lei C, Hu SJNSAfTA-EA Molecular mechanisms of resistance to the EGFR monoclonal antibody cetuximab. 2023: 13-27.

[6] Huang L, Fu L (2015). Mechanisms of resistance to EGFR tyrosine kinase inhibitors. Acta Pharm Sin B.

[7] Forde PM, Ettinger DS (2015). Managing acquired resistance in EGFR-mutated non-small cell lung cancer. Clin Adv Hematol Oncol.

[8] Visvader JE, Lindeman GJ (2012). Cancer stem cells: current status and evolving complexities. Cell Stem Cell.

[9] Willers H, Azzoli CG, Santivasi WL, Xia F (2013). Basic mechanisms of therapeutic resistance to radiation and chemotherapy in lung cancer. Cancer J.

[10] Shien K, Toyooka S, Yamamoto H, Soh J, Jida M, Thu KL (2013). Acquired resistance to EGFR inhibitors is associated with a manifestation of stem cell-like properties in cancer cells. Cancer Res.

[11] Corominas-Faja B, Oliveras-Ferraros C, Cuyas E, Segura-Carretero A, Joven J, Martin-Castillo B (2013). Stem cell-like ALDH(bright) cellular states in EGFR-mutant non-small cell lung cancer: a novel mechanism of acquired resistance to erlotinib targetable with the natural polyphenol silibinin. Cell Cycle.

[12] Arasada RR, Amann JM, Rahman MA, Huppert SS, Carbone DP (2014). EGFR blockade enriches for lung cancer stem-like cells through Notch3-dependent signaling. Cancer Res.

[13] Kelly K, Chansky K, Gaspar LE, Albain KS, Jett J, Ung YC (2008). Phase III trial of maintenance gefitinib or placebo after concurrent chemoradiotherapy and docetaxel consolidation in inoperable stage III non-small-cell lung cancer: SWOG S0023. J Clin Oncol.

[14] Goss GD, O'Callaghan C, Lorimer I, Tsao MS, Masters GA, Jett J (2013). Gefitinib versus placebo in completely resected non-small-cell lung cancer: results of the NCIC CTG BR19 study. J Clin Oncol.

[15] Eyler CE, Rich JN (2008). Survival of the fittest: cancer stem cells in therapeutic resistance and angiogenesis. J Clin Oncol.

[16] Maugeri-Sacca M, Bartucci M, De Maria R (2012). DNA damage repair pathways in cancer stem cells. Mol Cancer Ther.

[17] Pilie PG, Gay CM, Byers LA, O'Connor MJ, Yap TA (2019). PARP Inhibitors: Extending Benefit Beyond BRCA-Mutant Cancers. Clin Cancer Res.

[18] Robins HI, Zhang P, Gilbert MR, Chakravarti A, de Groot JF, Grimm SA (2016). A randomized phase I/II study of ABT-888 in combination with temozolomide in recurrent temozolomide resistant glioblastoma: an NRG oncology RTOG group study. J Neurooncol.

[19] Middleton MR, Friedlander P, Hamid O, Daud A, Plummer R, Falotico N (2015). Randomized phase II study evaluating veliparib (ABT-888) with temozolomide in patients with metastatic melanoma. Ann Oncol.

[20] Wang B, Chu D, Feng Y, Shen Y, Aoyagi-Scharber M, Post LE (2016). Discovery and Characterization of (8S,9R)-5-Fluoro-8-(4-fluorophenyl)-9-(1-methyl-1H-1,2,4-triazol-5-yl)-2,7,8,9-te trahydro-3H-pyrido[4,3,2-de]phthalazin-3-one (BMN 673, Talazoparib), a Novel, Highly Potent, and Orally Efficacious Poly(ADP-ribose) Polymerase-1/2 Inhibitor, as an Anticancer Agent. J Med Chem.

[21] Murai J, Huang SY, Renaud A, Zhang Y, Ji J, Takeda S (2014). Stereospecific PARP trapping by BMN 673 and comparison with olaparib and rucaparib. Mol Cancer Ther.

[22] Remon J, Besse B, Leary A, Bieche I, Job B, Lacroix L (2020). Somatic and Germline BRCA 1 and 2 Mutations in Advanced NSCLC From the SAFIR02-Lung Trial. JTO clinical and research reports.

[23] Schmitt J, Huang S, Goodfellow E, Williams C, Jean-Claude BJ (2020). Design and Synthesis of a Trifunctional Molecular System "Programmed" to Block Epidermal Growth Factor Receptor Tyrosine Kinase, Induce High Levels of DNA Damage, and Inhibit the DNA Repair Enzyme (Poly(ADP-ribose) Polymerase) in Prostate Cancer Cells. J Med Chem.

[24] Yacoub A, McKinstry R, Hinman D, Chung T, Dent P, Hagan MP (2003). Epidermal growth factor and ionizing radiation up-regulate the DNA repair genes XRCC1 and ERCC1 in DU145 and LNCaP prostate carcinoma through MAPK signaling. Radiat Res.

[25] Pfaffle HN, Wang M, Gheorghiu L, Ferraiolo N, Greninger P, Borgmann K (2013). EGFR-activating mutations correlate with a Fanconi anemia-like cellular phenotype that includes PARP inhibitor sensitivity. Cancer Res.

[26] Wu S, Gao F, Zheng S, Zhang C, Martinez-Ledesma E, Ezhilarasan R (2020). EGFR Amplification Induces Increased DNA Damage Response and Renders Selective Sensitivity to Talazoparib (PARP Inhibitor) in Glioblastoma. Clin Cancer Res.

[27] Kumar JP, Moses K (2001). EGF receptor and Notch signaling act upstream of Eyeless/Pax6 to control eye specification. Cell.

[28] Price JV, Savenye ED, Lum D, Breitkreutz A (1997). Dominant enhancers of Egfr in Drosophila melanogaster: genetic links between the Notch and Egfr signaling pathways. Genetics.

[29] Takebe N, Miele L, Harris PJ, Jeong W, Bando H, Kahn M (2015). Targeting Notch, Hedgehog, and Wnt pathways in cancer stem cells: clinical update. Nature reviews Clinical oncology.

[30] Baker AT, Zlobin A, Osipo C (2014). Notch-EGFR/HER2 Bidirectional Crosstalk in Breast Cancer. Frontiers in oncology.

[31] Schmitz S, Bindea G, Albu RI, Mlecnik B, Machiels JP (2015). Cetuximab promotes epithelial to mesenchymal transition and cancer associated fibroblasts in patients with head and neck cancer. Oncotarget.

[32] Fu W, Lei C, Yu Y, Liu S, Li T, Lin F (2019). EGFR/Notch Antagonists Enhance the Response to Inhibitors of the PI3K-Akt Pathway by Decreasing Tumor-Initiating Cell Frequency. Clin Cancer Res.

[33] Hu S, Fu W, Li T, Yuan Q, Wang F, Lv G (2017). Antagonism of EGFR and Notch limits resistance to EGFR inhibitors and radiation by decreasing tumor-initiating cell frequency. Science translational medicine.

[34] Arasada RR, Shilo K, Yamada T, Zhang J, Yano S, Ghanem R (2018). Notch3-dependent β-catenin signaling mediates EGFR TKI drug persistence in EGFR mutant NSCLC. Nature communications.

[35] Schmittgen TD, Livak KJJNp Analyzing real-time PCR data by the comparative CT method. 2008; 3: 1101-8.

[36] Ullman-Cullere MH, Foltz CJ (1999). Body condition scoring: a rapid and accurate method for assessing health status in mice. Laboratory animal science.

[37] Sullivan JP, Spinola M, Dodge M, Raso MG, Behrens C, Gao B (2010). Aldehyde dehydrogenase activity selects for lung adenocarcinoma stem cells dependent on notch signaling. Cancer Res.

[38] Medema JP (2013). Cancer stem cells: the challenges ahead. Nature cell biology.

[39] Ma S, Chan KW, Lee TK, Tang KH, Wo JY, Zheng BJ (2008). Aldehyde dehydrogenase discriminates the CD133 liver cancer stem cell populations. Molecular cancer research: MCR.

[40] Konishi J, Kawaguchi KS, Vo H, Haruki N, Gonzalez A, Carbone DP (2007). Gamma-secretase inhibitor prevents Notch3 activation and reduces proliferation in human lung cancers. Cancer Res.

[41] RL Y, T J, DA E, G C, W Z, L F (2005). Epithelial versus mesenchymal phenotype determines in vitro sensitivity and predicts clinical activity of erlotinib in lung cancer patients. Clinical Cancer Research.

[42] Kraus WLJMc PARPs and ADP-ribosylation: 50 years… and counting. 2015; 58: 902-10.

[43] Andrabi SA, Dawson TM, Dawson VL (2008). Mitochondrial and nuclear cross talk in cell death: parthanatos. Annals of the New York Academy of Sciences.

[44] Chiu LY, Ho FM, Shiah SG, Chang Y, Lin WW (2011). Oxidative stress initiates DNA damager MNNG-induced poly(ADP-ribose)polymerase-1-dependent parthanatos cell death. Biochemical pharmacology.

[45] D'Amours D, Desnoyers S, D'Silva I, Poirier GG (1999). Poly(ADP-ribosyl)ation reactions in the regulation of nuclear functions. The Biochemical journal.

[46] Murai J, Huang SY, Das BB, Renaud A, Zhang Y, Doroshow JH (2012). Trapping of PARP1 and PARP2 by Clinical PARP Inhibitors. Cancer Res.

[47] Krishnakumar R, Gamble MJ, Frizzell KM, Berrocal JG, Kininis M, Kraus WL (2008). Reciprocal binding of PARP-1 and histone H1 at promoters specifies transcriptional outcomes. Science.

[48] Luo X, Kraus WL (2012). On PAR with PARP: cellular stress signaling through poly(ADP-ribose) and PARP-1. Gene Dev.

[49] Schiewer MJ, Knudsen KE (2014). Transcriptional roles of PARP1 in cancer. Molecular cancer research: MCR.

[50] Kantidze OL, Velichko AK, Luzhin AV, Petrova NV, Razin SV (2018). Synthetically Lethal Interactions of ATM, ATR, and DNA-PKcs. Trends in cancer.

[51] Lin KH, Rutter JC, Xie A, Pardieu B, Winn ET, Bello RD (2020). Using antagonistic pleiotropy to design a chemotherapy-induced evolutionary trap to target drug resistance in cancer. Nature genetics.

[52] Nowsheen S, Cooper T, Stanley JA, Yang ES (2012). Synthetic Lethal Interactions between EGFR and PARP Inhibition in Human Triple Negative Breast Cancer Cells. Plos One.

[53] Mazzarella L, Guida A, Curigliano G (2018). Cetuximab for treating non-small cell lung cancer. Expert Opin Biol Ther.

