# Supplementary material for: Bispecific antibodies targeting EGFR/Notch enhance the response to talazoparib by decreasing tumour-initiating cell frequency
Source: Theranostics. 2023 Jun 26;13(11):3641–54. doi: 10.7150/thno.82144 (PMC10334837; doi:10.7150/thno.82144)
Supplement: Supplementary file 1 — Supplementary figures and tables. [file thnov13p3641s1.pdf]

Supplementary Figures

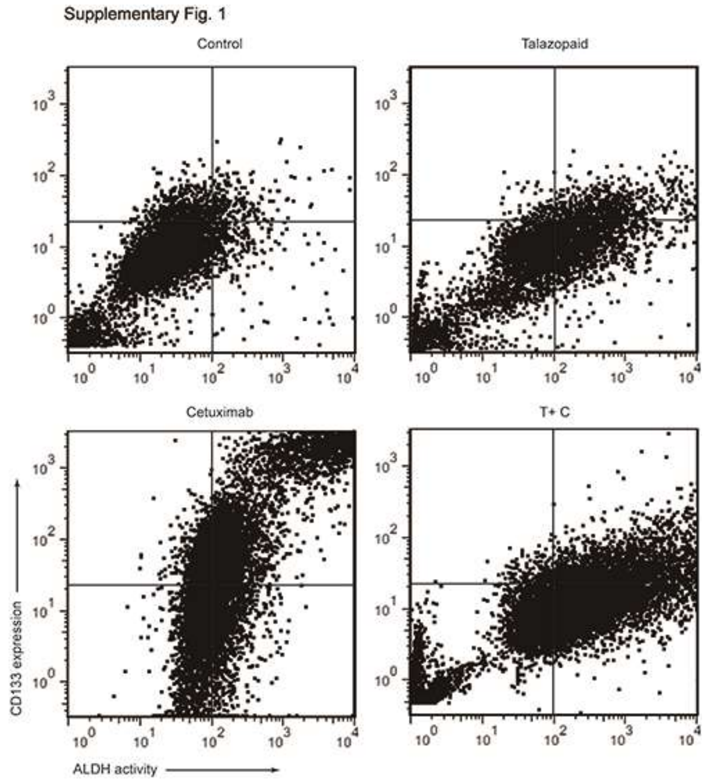

Figure S1. EGFR and PARP blockade increases the CSC subpopulation of NSCLC cells. An example of HCC827 cells treated with cetuximab, talazoparib, or cetuximab plus talazoparib for 4 days, and the resulting cells were analysed by fluorescence-activated cell sorting (FACS). Results shown represent six independent experiments.

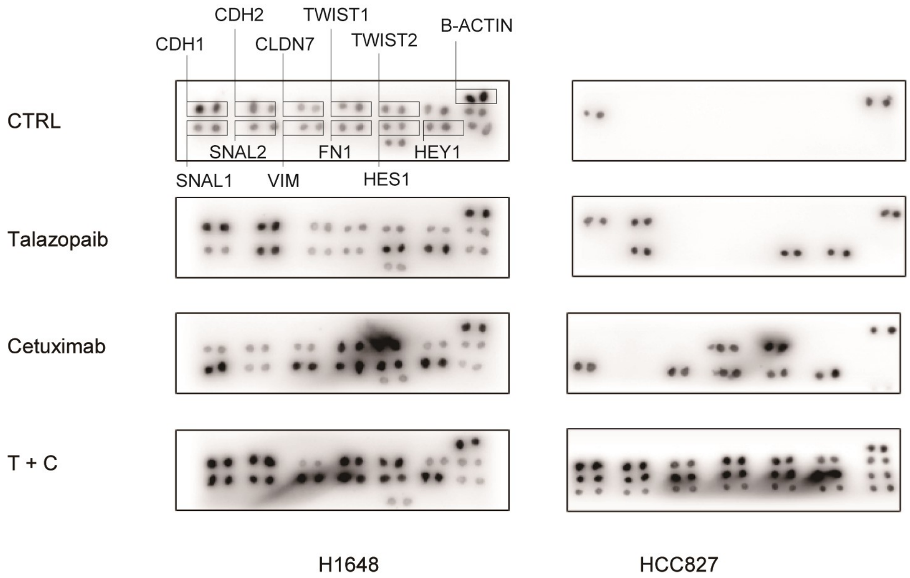

Figure S2. EGFR and PARP blockade promote EMT. H1648 or HCC827 cells were treated with different drugs on Day 1. Select protein expression was determined by a custom protein array.

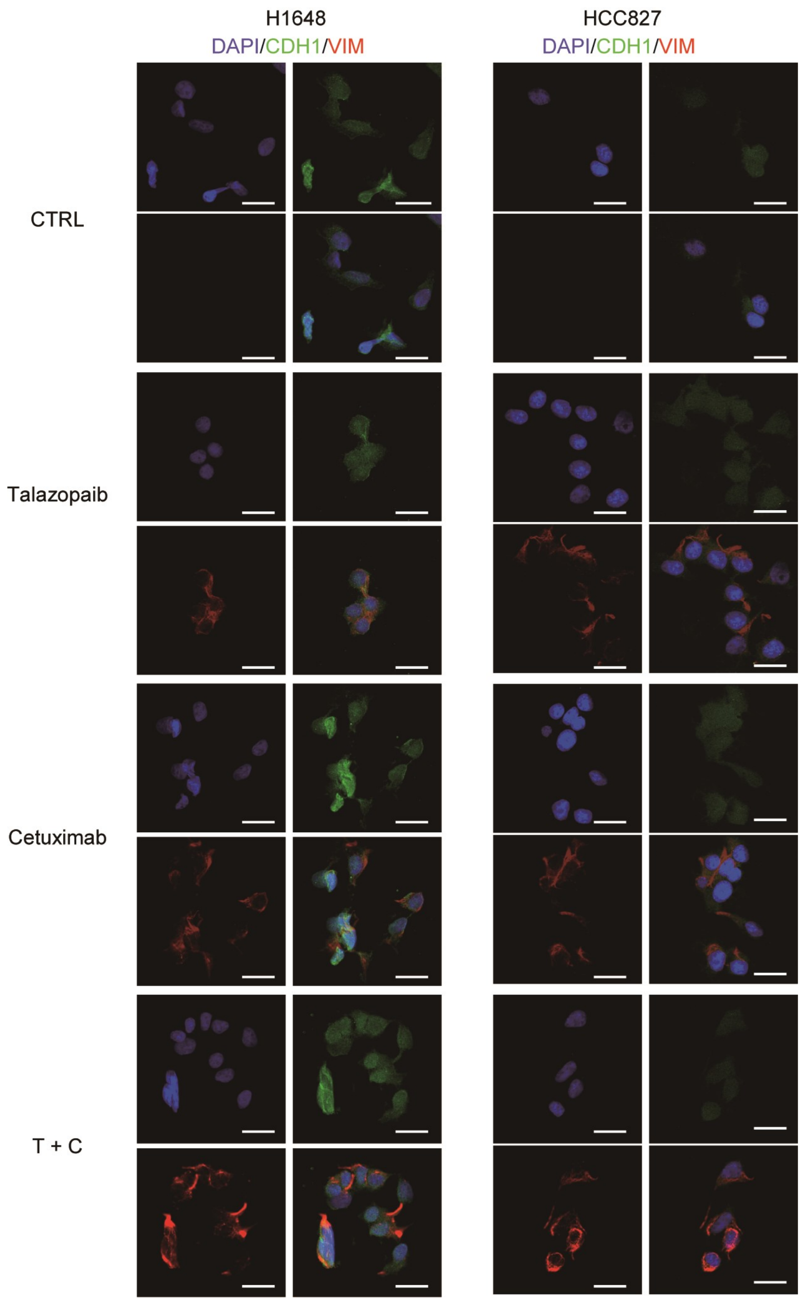

Figure S3. EGFR and PARP blockade promote EMT. Immunofluorescence analysis of H1648 cells (left) and HCC827 cells (right) after treatment of different antibodies. The experiments were repeated independently three times with similar results. Scale bars= 50 μm.

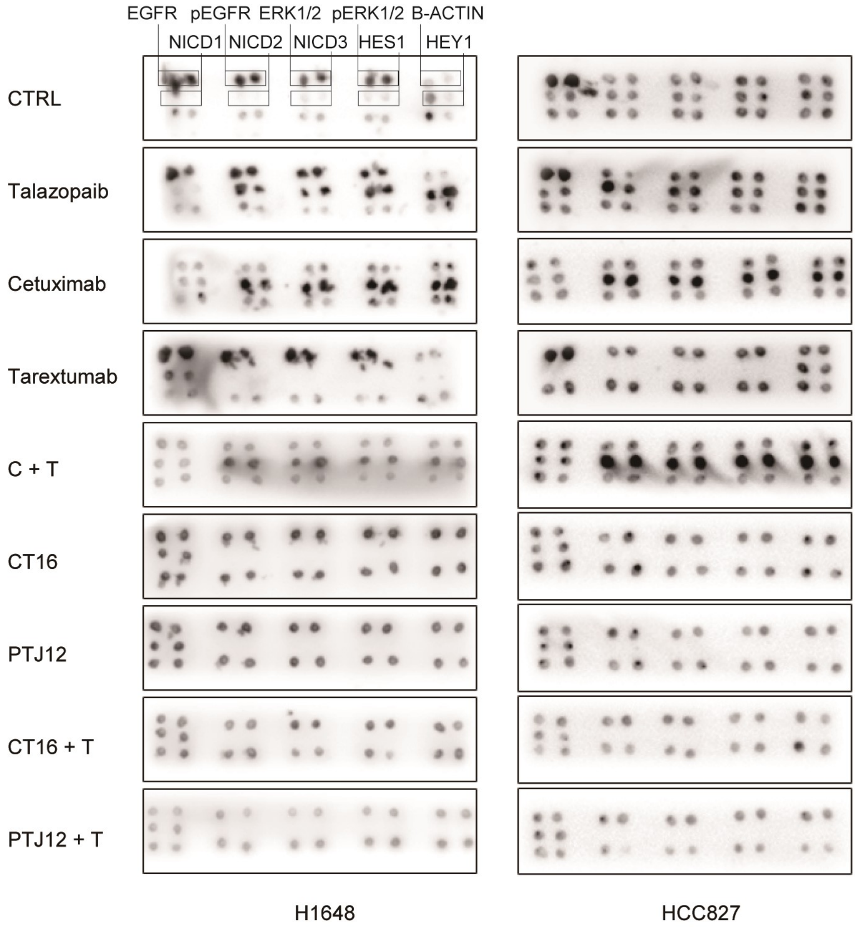

Figure S4. Effect of different antibodies and dugs on EGFR and Notch signaling. H1648 or HCC827 cells were treated with different drugs. Select protein expression was determined by a custom protein array.

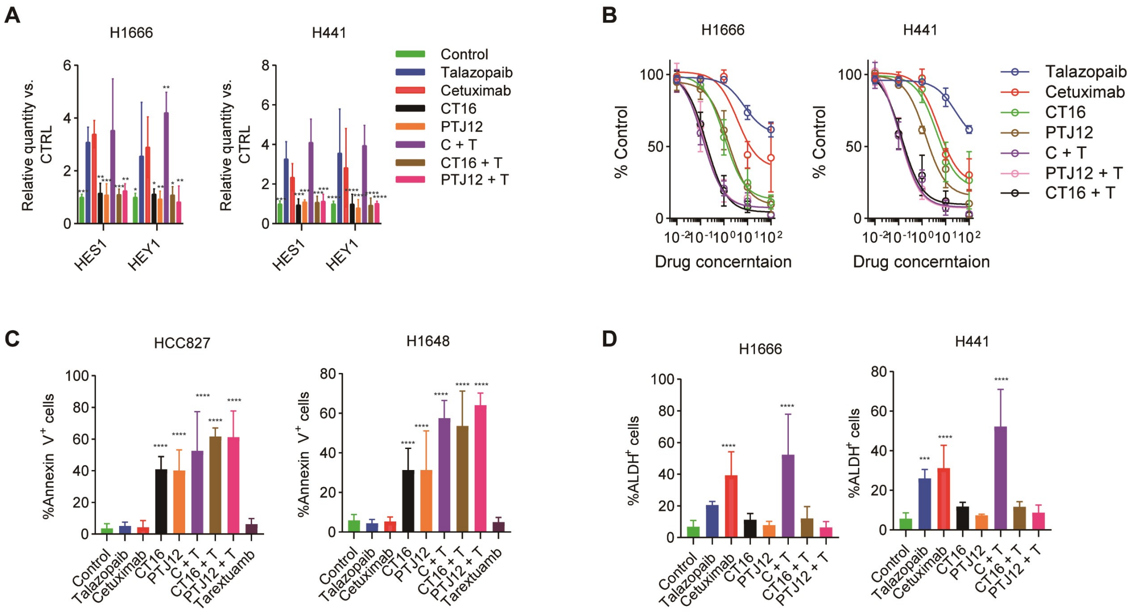

Figure S5. In vitro characterization of EGFR, PARP and Notch blockers. A, Cells were treated with the drugs specified in the figure, and selected gene expression was determined by qPCR analysis. Gene expression was normalized to the housekeeping gene b-actin and is expressed as the fold change compared to control cells. **B**, NSCLC cells were treated with increasing concentrations of the indicated drugs [talazoparib (μM), antibodies (μg/ml)]. Cell proliferation relative to an untreated control was measured after 4 days using alamarBlue staining. **C**, HCC827 and H1648 cells were treated with different drugs to induce apoptosis/necrosis, as assessed by annexin-V staining. **D.** NSCLC cells were treated with different drugs for 4 days, and the resulting cells were analysed via FACS. Data are presented as the mean ± s.d. of six independent biological replicates (A-D). *P* values were obtained using two-way ANOVA followed by a Bonferroni post-test (A, B) or one-way ANOVA followed by a Tukey post-test (C, D). *, $P < 0.05$; **, $P < 0.01$; ***, $P < 0.001$; ****, $P < 0.0001$; versus CTRL.

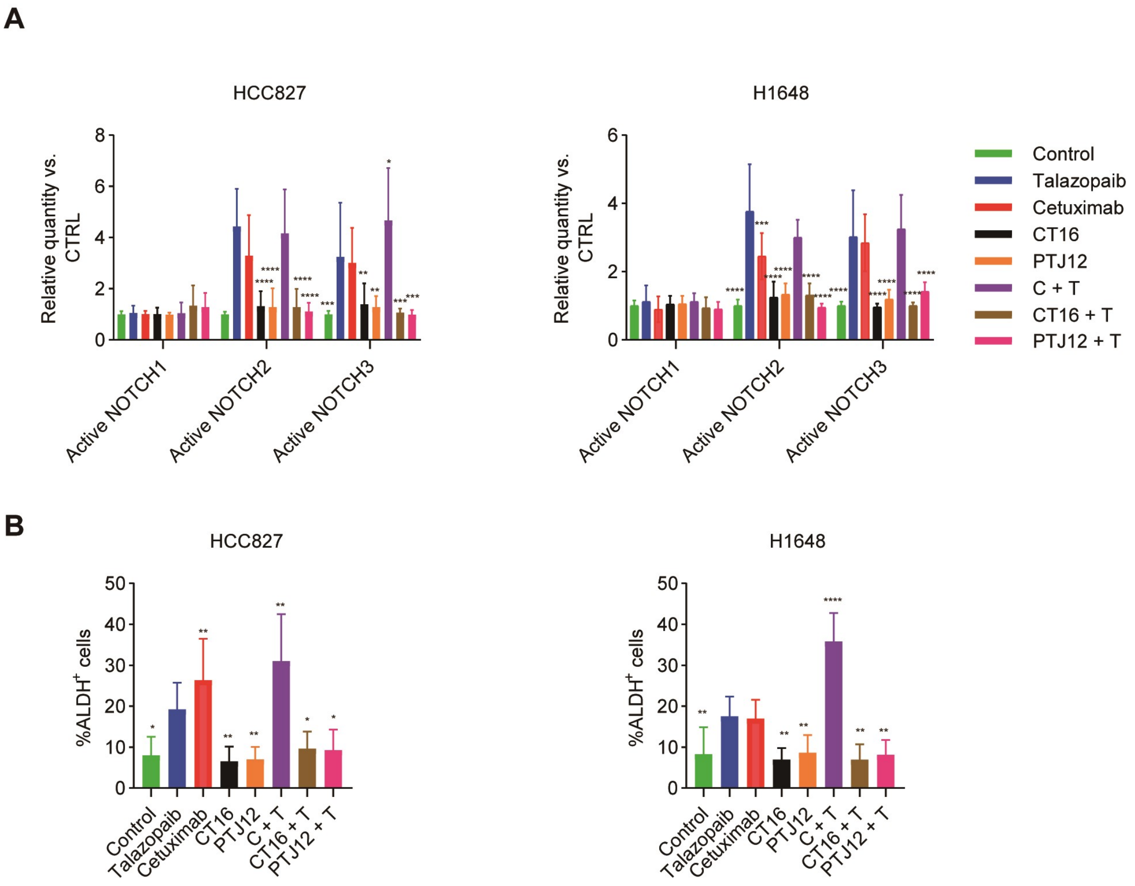

Figure S6. In vitro characterization of Notch activation and ALDH+ subsets after different treatment. a, HCC827 and H1648 tumours were harvest and active Notch receptors were measured by ELISA in 6 independent experiments. b. ALDH + cancer cells from tumour of treated mice was measured by flow cytometry. Data are presented as the mean ± s.d. of six independent biological replicates (a-d). *P* values were obtained using two-way ANOVA followed by a Bonferroni post-test (a) or one-way ANOVA followed by a Tukey post-test (b). *, $P < 0.05$; **, $P < 0.01$; ***, $P < 0.001$; ****, $P < 0.0001$; versus Talazopaib group.

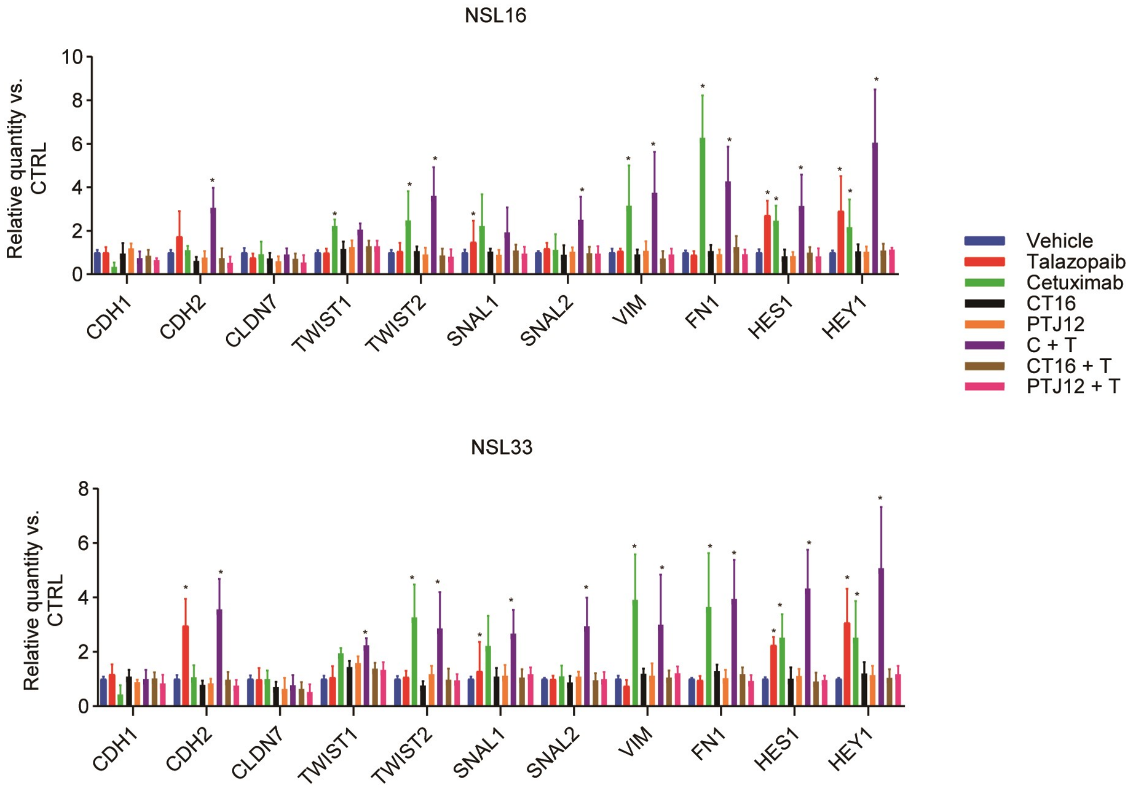

Figure S7. EMT gene expression after different treatment. qPCR analysis was conducted to determine the expression of selected genes in NSL16 and NSL33 tumours subjected to different treatments. Gene expression was normalized to the housekeeping gene b-actin and is expressed as the fold change compared with the vehicle group. Data are presented as the mean ± s.d.. *P* values were obtained using two-way ANOVA followed by a Bonferroni post-test; *, $P < 0.05$ versus CTRL.

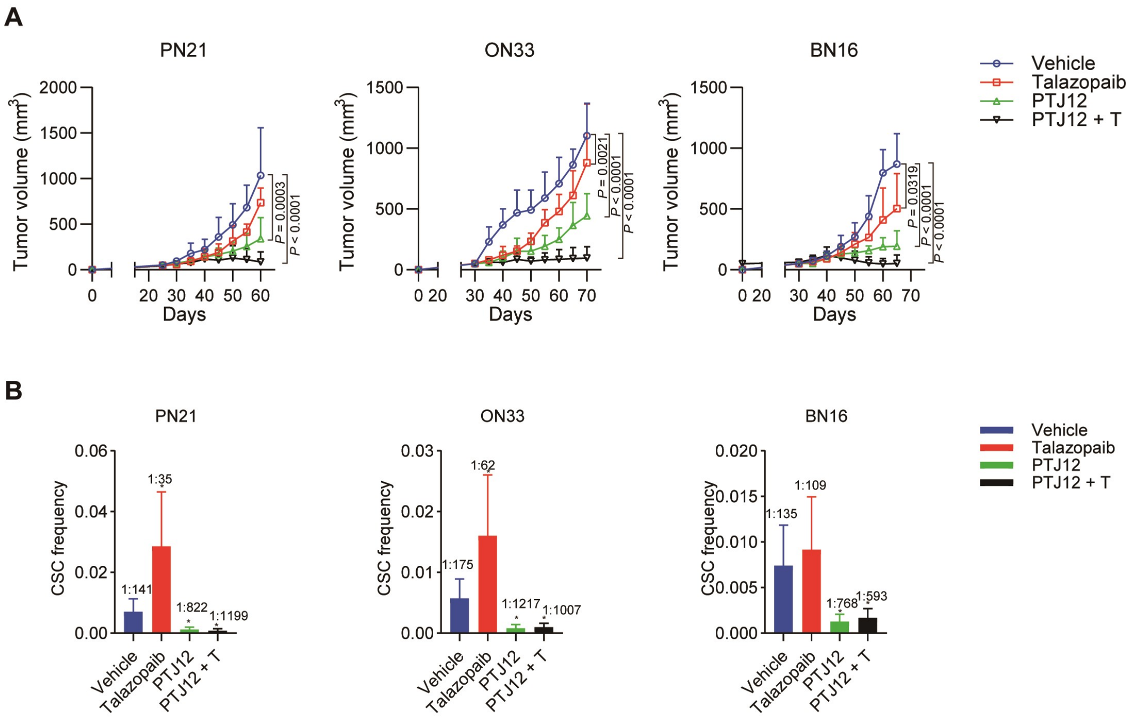

Figure S8. Activity of PTJ12 plus talazoparib on pancreatic, ovarian, and breast xenograft tumours. Antitumour effect of PTJ12 plus talazoparib on PN21 (pancreatic tumour), ON33 (serous ovarian tumour) and BN16 (triple negative breast tumour). The data are presented as the means ± SEM; n = 8 animals per group. *P < 0.05 versus vehicle, #P < 0.05 versus GDC-0941 by two-way ANOVA followed by Bonferroni post-test comparisons. **(B)** The effect of different treatments on CSC frequency on PN21, ON33 and BN16 tumours at the end of the in vivo study. *P* values were obtained using two-way ANOVA followed by a Bonferroni post-test (a) or using one-way ANOVA followed by a Tukey post-test (b); *, *P* < 0.05 versus vehicle.

Supplementary Tables

Table S1. IC50 values.

Cell line	Talazoparib (μM)	Cetuximab (μg/ml)
H1792	0.52 ± 0.18	-
H23	1.43 ± 0.46	-
A549	2.10 ± 0.17	32.86 ± 4.64
H1944	4.79 ± 1.89	-
H2122	4.83 ± 1.03	39.21 ± 1.27
H2405	7.01 ± 2.65	-
H2030	7.43 ± 1.31	-
H2009	11.80 ± 0.65	-
H2291	11.86 ± 2.89	-
Calu-3	13.78 ± 1.08	3.35 ± 0.12
H1568	15.05 ± 3.02	7.98 ± 0.41
H1355	16.15 ± 1.91	-
H3255	17.47 ± 1.16	9.15 ± 0.66
H1666	20.22 ± 1.16	10.46 ± 0.79
H1975	22.18 ± 3.38	-
HCC-827	32.76 ± 2.22	10.90 ± 1.66
H1648	34.57 ± 5.68	8.22 ±1.66
H441	51.23 ± 10.82	28.54 ± 4.49
H358	145.44 ± 19.87	5.82 ± 0.60

H1838	163.30 ± 15.88	1.58 ± 0.
H322	145.43 ± 19.87	19.42 ± 0.81

Table S2. Tumor growth inhibitory effects of different drugs in murine xenograft models.

Model	cetuximab	talazoparib	CT16	PTJ12	CT16+T	PTJ12+T	C+T
HCC827	+++	+	+++	+++	+++	+++	+++
827C	−	+	−	−	−	−	−
827T	++	−	++	++	++	++	++
H1648	++	+	+++	+++	+++	+++	+++
1648C	−	+	−	−	−	−	−
1648T	+	−	+	+	+	+	+

Mice were treated with 20 mg/kg cetuximab, 80 mg/kg talazoparib, 20 mg/kg CT16, 20mg/kg PTJ12, the combination of 20mg/kg antibodies plus 40 mg/kg talazoparib once a week for 4 cycles. Initial dose was a 2x loading dose for all treatments. Percent of tumor growth inhibition (TGI) was calculated for each study based on the last day of study in which the majority of mice remained in the vehicle group. TGI below 25% is indicated as −, TGI between 25-50 % is indicated as +, TGI between 51-75% is indicated as ++, and TGI of 76% and above as +++.

Table S2. Tumor growth inhibitory effect of different drugs in PDX models.

Sample	C*	T	H	EGFR IHC score	EGFR status	KRAS status
NSL4	−	++	SCC	250	wild-type	wild-type
NSL9	−	+	LCC	150	wild-type	wild-type
NSL11	++	+	LCC	200	wild-type	wild-type
NSL16	++	+	SCC	125	wild-type	wild-type
NSL27	−	+	SCC	300	wild-type	Q61H
NSL28	−	−	SCC	0	wild-type	wild-type
NSL33	++	+	LCC	150	wild-type	wild-type
NSL41	−	−	LCC	290	wild-type	wild-type
NSL44	−	+++	AC	195	wild-type	wild-type
NSL46	−	−	SCC	275	E746_delA750	wild-type

* Mice were treated with 20 mg/kg cetuximab, 80 mg/kg talazoparib, 20 mg/kg CT16, 20mg/kg PTJ12, the combination of 20mg/kg antibodies plus 40 mg/kg talazoparib once a week for 4 cycles. Initial dose was a 2x loading dose for all treatments. Percent of tumor growth inhibition (TGI) was calculated for each study based on the day of study in which the mean volume of tumor in the vehicle group reach 400 mm^3. TGI below 25% is indicated as −, TGI between 25-50 % is indicated as +, TGI between 51-75% is indicated as ++, and TGI of 76% and above as +++. *C, Cetuximab; T, talazoparib; H, Histological subtype.

Table S4. Multiple comparisons test related to Figure 5b

Model	Multiple comparisons	P Value
NSL11	Vehicle vs. Talazopaib	0.9168
	Vehicle vs. Cetuximab	0.8736
	Vehicle vs. CT16	0.0003
	Vehicle vs. PTJ12	0.0017
	Vehicle vs. C + T	>0.9999
	Vehicle vs. CT16 + T	<0.0001
	Vehicle vs. PTJ12 + T	<0.0001
	Talazopaib vs. Cetuximab	>0.9999
	Talazopaib vs. CT16	0.0205
	Talazopaib vs. PTJ12	0.0931
	Talazopaib vs. C + T	0.9849
	Talazopaib vs. CT16 + T	<0.0001
	Talazopaib vs. PTJ12 + T	<0.0001
	Cetuximab vs. CT16	0.0303
	Cetuximab vs. PTJ12	0.1255
	Cetuximab vs. C + T	0.9733
	Cetuximab vs. CT16 + T	0.0001
	Cetuximab vs. PTJ12 + T	<0.0001
	CT16 vs. PTJ12	0.9989
	CT16 vs. C + T	0.0002
	CT16 vs. CT16 + T	0.0409
	CT16 vs. PTJ12 + T	0.0006
	PTJ12 vs. C + T	0.0020
	PTJ12 vs. CT16 + T	0.0452
	PTJ12 vs. PTJ12 + T	0.0030
	C + T vs. CT16 + T	<0.0001
	C + T vs. PTJ12 + T	<0.0001
	CT16 + T vs. PTJ12 + T	0.9160
NSL16	Vehicle vs. Talazopaib	0.1014
	Vehicle vs. Cetuximab	0.0552
	Vehicle vs. CT16	0.0008
	Vehicle vs. PTJ12	0.0016
	Vehicle vs. C + T	0.0154
	Vehicle vs. CT16 + T	<0.0001
	Vehicle vs. PTJ12 + T	<0.0001
	Talazopaib vs. Cetuximab	>0.9999
	Talazopaib vs. CT16	0.7208
	Talazopaib vs. PTJ12	0.7503
	Talazopaib vs. C + T	0.9903
	Talazopaib vs. CT16 + T	<0.0001
	Talazopaib vs. PTJ12 + T	<0.0001
	Cetuximab vs. CT16	0.8775
	Cetuximab vs. PTJ12	0.8845
	Cetuximab vs. C + T	0.9991
	Cetuximab vs. CT16 + T	<0.0001
	Cetuximab vs. PTJ12 + T	0.0001

	CT16 vs. PTJ12	>0.9999
	CT16 vs. C + T	0.9978
	CT16 vs. CT16 + T	<0.0001
	CT16 vs. PTJ12 + T	0.0018
	PTJ12 vs. C + T	0.9962
	PTJ12 vs. CT16 + T	0.0045
	PTJ12 vs. PTJ12 + T	0.0351
	C + T vs. CT16 + T	0.0003
	C + T vs. PTJ12 + T	0.0033
	CT16 + T vs. PTJ12 + T	0.9979
NSL33	Vehicle vs. Talazopaib	0.1112
	Vehicle vs. Cetuximab	0.0338
	Vehicle vs. CT16	<0.0001
	Vehicle vs. PTJ12	<0.0001
	Vehicle vs. C + T	0.0014
	Vehicle vs. CT16 + T	<0.0001
	Vehicle vs. PTJ12 + T	<0.0001
	Talazopaib vs. Cetuximab	0.9997
	Talazopaib vs. CT16	0.2244
	Talazopaib vs. PTJ12	0.1042
	Talazopaib vs. C + T	0.7742
	Talazopaib vs. CT16 + T	<0.0001
	Talazopaib vs. PTJ12 + T	<0.0001
	Cetuximab vs. CT16	0.5414
	Cetuximab vs. PTJ12	0.3287
	Cetuximab vs. C + T	0.9643
	Cetuximab vs. CT16 + T	0.0006
	Cetuximab vs. PTJ12 + T	<0.0001
	CT16 vs. PTJ12	>0.9999
	CT16 vs. C + T	0.9942
	CT16 vs. CT16 + T	0.0320
	CT16 vs. PTJ12 + T	0.0006
	PTJ12 vs. C + T	0.9605
	PTJ12 vs. CT16 + T	0.0284
	PTJ12 vs. PTJ12 + T	0.0002
	C + T vs. CT16 + T	0.0229
	C + T vs. PTJ12 + T	0.0016
	CT16 + T vs. PTJ12 + T	0.7502
